# A molecular and conchological dissection of the “scaly” *Georissa* of Malaysian Borneo (Gastropoda, Neritimorpha, Hydrocenidae)

**DOI:** 10.3897/zookeys.773.24878

**Published:** 2018-07-09

**Authors:** Mohd Zacaery Khalik, Kasper Hendriks, Jaap J. Vermeulen, Menno Schilthuizen

**Affiliations:** 1 Naturalis Biodiversity Center, Vondellaan 55, 2332 AA Leiden, The Netherlands; 2 Institute of Biology Leiden, Faculty of Science, Leiden University, 2333 BE Leiden, The Netherlands; 3 Department of Zoology, Faculty of Resource Science and Technology, Universiti Malaysia Sarawak, 94300 Kota Samarahan, Sarawak, Malaysia; 4 Groningen Institute for Evolutionary Life Sciences, Faculty of Mathematics and Natural Sciences, University of Groningen, 9747 AG Groningen, The Netherlands; 5 JK Art and Science, Lauwerbes 8, 2318 AT Leiden, The Netherlands; 6 Institute for Tropical Biology and Conservation, Universiti Malaysia Sabah, Jalan UMS, 88400 Kota Kinabalu, Sabah, Malaysia

**Keywords:** Gastropods, land snail, limestone karst, Malaysian Borneo, micro-computed tomography, Sabah, Sarawak, species delimitation

## Abstract

The Bornean hydrocenids have so far been understudied compared to other non-pulmonate snails in this region. In the present study, we review a first group of minute land snail species belonging to the genus *Georissa* (Gastropoda, Hydrocenidae) from Malaysian Borneo. This group is restricted to the species with conspicuous scale-like sculpture on the shell. Based on materials from recent fieldwork, museums, and personal collections, Malaysian Borneo hydrocenids are more complex and diverse in shell characters than previously anticipated. Here, a molecular, conchological, and biogeographic study of this “scaly group” is presented. We recognise 13 species of which six are new to science, namely *Georissa anyiensis* **sp. n.**, *Georissa muluensis* **sp. n.**, *Georissa bauensis* **sp. n.**, *Georissa silaburensis* **sp. n.**, *Georissa kinabatanganensis* **sp. n.**, and *Georissa sepulutensis* **sp. n.**

## Introduction

Over the past 25 years, the microsnail fauna of karst habitats in South East Asia has enjoyed an ongoing surge of attention. Detailed conchological and molecular studies in this region have revealed high allopatric and sympatric diversity (e.g., Liew et al. 2014, Rundell 2008, Tongkerd et al. 2004), which has opened up this fauna for work in the fields of community ecology (Schilthuizen et al. 2005, Schilthuizen 2011), speciation (Schilthuizen et al. 2006, Schilthuizen et al. 2012), and conservation biology (Clements et al. 2006, Clements et al. 2008, Schilthuizen et al. 2005). Although several families of non-pulmonate snails have featured prominently in these studies (in particular the Diplommatinidae and other cyclophoroids), the family Hydrocenidae (Neritimorpha) has so far been understudied. In this paper, we make a start with a first conchological and molecular characterisation of a surprisingly diverse group of species in the genus *Georissa* Blanford, 1864.

The genus *Georissa* Blanford, 1864 is characterised by a calcareous, rounded to ovate concentric, paucispiral operculum, with a calcareous peg emerging from the inner surface (Bandel 2008, Thompson and Dance 1983, Vermeulen et al. 2015). The shell is small, dextral, conical, and frequently presents conspicuous radial and spiral sculpture. The studies by Thompson and Dance (1983) and Vermeulen et al. (2015) showed that the Bornean *Georissa* are between 0.7 and 4.0 mm in adult shell height. The protoconch is usually distinctly hemi-spherically shaped, distinct in microsculpture and distinguishable from the post-embryonic whorls. The internal walls (some would refer these as septa) are resorbed, and the remaining wall ends more than one whorl before reaching the aperture; resorption also leads to excavation of the columella (Thompson and Dance 1983, Bandel 2008). The evolutionary causes for this internal shell restructuring remain to be studied. The snails are often found in moderate to high densities on rocks, especially limestone rocks, where they apparently forage moss, algae, and lichens (Berry 1966). Cave-adapted species may forage on bacterial films (Schilthuizen et al. 2012).

Previous taxonomic treatments of Bornean *Georissa* (Godwin-Austen 1889, Gredler 1902, Haase and Schilthuizen 2007, Smith 1893, 1895, Thompson and Dance 1983, van Benthem-Jutting 1966, Vermeulen and Junau 2007, Vermeulen et al. 2015) revealed that shell shape and size, as well as sculptural patterns on the whorls are important characters for species delimitation. Given the small size of these shells, great benefits can be had from the use of scanning electron microscopy and X-ray microtomography, which are able to show detailed microscopic sculpture patterns and the inner part of the shell.

Since the overview presented by Thompson and Dance (1983), no revisions have been made for the Bornean *Georissa*, although recently, several new Bornean *Georissa* have been described, i.e., *Georissa filiasaulae* Haase & Schilthuizen, 2007, *Georissa pachysoma* Vermeulen & Junau, 2007, *Georissa leucococca* Vermeulen et al., 2015 and *Georissa nephrostoma* Vermeulen et al., 2015. Our new studies of the *Georissa* of Malaysian Borneo reveal additional, previously unrecognized diversity, which warrants a series of revisions of the various species groups. In the present paper, we first address a group of species that we here call the “scaly group”, chiefly consisting of species with conspicuous scale-like sculpture on the shell.

We present detailed species descriptions for a total of 13 Bornean *Georissa* from the “scaly group”, of which six species are new to science, namely: *Georissa anyiensis* sp. n., *Georissa muluensis* sp. n., *Georissa bauensis* sp. n., *Georissa silaburensis* sp. n., *Georissa kinabatanganensis* sp. n., and *Georissa sepulutensis* sp. n.

## Materials and methods

### Materials and fieldwork

We examined collection material from:

**RMNH** Naturalis Biodiversity Center (previously collection from Rijksmuseum van Natuurlijke Historie), Leiden,

**ZMA** Naturalis Biodiversity Center (previously collection from Zoological Museum of Amsterdam), Leiden,

**NHMUK** Natural History Museum, London,

**BORN** Universiti Malaysia Sabah,

**MZU** Zoology Museum, Universiti Malaysia Sarawak, and

**JJV** Jaap Vermeulen (personal collection).

In addition to these available data, we did fieldwork at limestone outcrops in Malaysian Borneo between September 2015 and May 2017. Manual searches were carried out to collect living and empty shells of *Georissa* on limestone walls and rocks, loose organic matter, and on/under living leaves. The living *Georissa* were directly stored in sample tubes containing ~96% ethanol. Ca. 5 litres of soil and leaf litter were sampled at each sampling location to collect empty shells by flotation (Vermeulen and Whitten 1998). The holotypes, paratypes and all of the collected materials were deposited at the Zoology Museum (Universiti Malaysia Sarawak, Kota Samarahan, Sarawak, Malaysia), Borneensis Collection (Universiti Malaysia Sabah, Kota Kinabalu, Sabah, Malaysia), and Naturalis Biodiversity Centre (Leiden, The Netherlands).

### Morphological analysis

**Microscopy**. Shells were observed with a Zeiss SteREO Microscope Discovery V20. The images of examined individuals were captured by AxioCamMRc5, Zeiss PlanApo S 1.0× FWD 60.0mm lenses. A complementary software of the camera AxioVision Special Edition 64-bit version 4.9.1.0 was used for shell measurements, namely, shell height, shell width, aperture height, and aperture width, at 30–60× magnification. The measurements of “scaly” *Georissa* were carried out following the shell measurement method of Vermeulen and Whitten 1998. **Scanning electron microscopy**. A representative adult shell for each species was cleaned using sodium hypochloride, dried, and sputter-coated with Pd/Pt coating agent before detailed examination with a JEOL JSM-6480LV scanning electron microscope (SEM). We obtained SEM images of the entire shell in top view and apertural view (including clear view of the sculpture), side and top views of the protoconch and the spire. **Micro-computed tomography**. The micro-computed tomography (µCT) scanning was carried out with an Xradia 520 Versa X-ray Microscope using accompanying software Zeiss Xradia Versa (11.1.6315). The X-ray images from the scanning (ca. 950 layers of images in TIF format) were reconstructed into composite 3D images of the shells using software Scout-and-Scan^TM^ Control System Reconstructor (11.1.5707.17179). All shell materials were scanned in air medium at 80/7 voltage/power (kW/P) using objective lens unit 4 in 180° rotation. Detailed scanning parameters for each species are summarized in Suppl. material 1. We used reconstructed 3D images of representative adult shells of each species from µCT scanned data to examine the internal characters, including the operculum and its peg. We conducted 3D image reconstruction to preserve the original structure of the shells and avoiding unintentional shell destruction. The 3D image analysis of the shells was carried out with Avizo ver. 9.2.0, FEI Company.

### Molecular analysis

**DNA extraction**. Genomic DNA was extracted from 127 individuals of *Georissa* using the Qiagen DNeasy Blood and Tissue kit, following the manufacturer’s protocol. Prior to the DNA extraction, the shells were removed and the entire soft tissue was used in the DNA extraction procedure. **DNA amplification**. We amplified two mtDNA regions, namely 16S and CO1. DNA amplifications were conducted on a BIO-RAD C1000 Touch Thermal Cycler. For the 16S gene, a fragment of 422-464 bp was amplified using primer pair LR-J-12887 5’-CCGGTCTGAACTCAGATCACGT-3’ (forward) and LR-N-13398 5’-CGCCTGTTTAACAAAAAACAT-3’ (reverse) (Schilthuizen et al. 2005) in 25.0 µL reaction volume, containing: 1.0 µL undiluted DNA template, 15.0 µL mQ (milli-Q, ultrapure water), 2.5 µL PCR chlorine buffer 10×, 2.5 µL MgCl_2_ 25.0 mM, 0.25 µL BSA 100 mM, 1.0 µL forward primer 10 pmol/µL, 1.0 µL reverse primer 10 pmol/µL, 1.5 µL dNTPs 2.5 mM, and 0.25 µL Taq 5.0 U/ µL. The amplification was carried out with the following cycling protocol: initial denaturation at 95 °C for 5 min, 36 cycles (of denaturation at 95 °C for 30 s, annealing at 52 °C for 30 s, extension at 72 °C for 1 min), and a final extension at 72 °C for 5 min. A 546-603 bp fragment of CO1 was amplified using primer pair LCO1490 5’-GGTCAACAAATCATAAAGATATTGG-3’ (forward) and HCO2198 5’-TAAACTTCAGGGTGACCAAAAAATCA-3’ (reverse) (Folmer et al. 1994) in 25.0 µL reaction volume, containing: 1.0 µL DNA template, 16.8 µL mQ, 2.5 µL PCR chlorine buffer 10×, 1.0 µL MgCl_2_ 25.0 mM, 1.0 µL BSA 100 mM, 1.0 µL forward primer 10 pmol/µL, 1.0 µL reverse primer 10 pmol/µL, 0.5 µL dNTPs 2.5 mM, and 0.25 µL Taq 5.0 U/µL. The amplification was carried out with the following cycling protocol: initial denaturation at 94 °C for 4 min, 40 cycles (of denaturation at 94 °C for 15 s, annealing at 50 °C for 30 s, extension at 72 °C for 40 s), and a final extension at 72 °C for 5 min. The unsuccessful amplication of CO1 and 16S genes were excluded in further phylogenetic analysis that used concatenated sequence alignment of both genes. **Sequencing**. The PCR products were then Sanger sequenced in both directions at BaseClear B.V. (Leiden, The Netherlands) on the ABI3730XL sequencer from Life Technologies. All new 16S mtDNA sequences used in this study were deposited in GenBank (https://www.ncbi.nlm.nih.gov/genbank/) and CO1 mtDNA sequences were deposited in GenBank via BOLD (http://boldsystems.org/), under accession numbers as listed in Table 1.

**Table 1.** List of specimens used in molecular analyses.

No.	Species	Voucher No.	Species name_sequence origin_locationTown/District/Division, State. GPS coordinate	GenBank Accession No.	
				16S	CO1
1	*Georissa gomantongensis* Smith, 1893	BOR/MOL 7389	G.gomantongensis_KPH01833.01_KinabatanganKinabatangan Valley, Sabah. 05°30.913'N, 118°16.889'E	MG982259	MH033876
2	*Georissa gomantongensis* Smith, 1893	BOR/MOL 7389	G.gomantongensis_KPH01833.02_KinabatanganKinabatangan Valley, Sabah. 05°30.913'N, 118°16.889'E	MG982260	MH033875
3	*Georissa saulae* (van Benthem Jutting, 1966)	BOR/MOL 2663-2667 (Schilthuizen et al. 2012)	G.saulae_AY547385_SinobangBatu Sinobang, Sabah. 04°48.040'N, 116°37.035'E	AY547385	n.a.
4	*Georissa saulae* (van Benthem Jutting, 1966)	BOR/MOL 2663-2667 (Schilthuizen et al. 2012)	G.saulae_hapA_AY547380_SanaronSepulut Valley, Batu Sanaron, Sabah. 04°42.052'N, 116°36.016'E	AY547380	n.a.
5	*Georissa saulae* (van Benthem Jutting, 1966)	BOR/MOL 2663-2667 (Schilthuizen et al. 2012)	G.saulae_hapB_AY547381_SanaronSepulut Valley, Batu Sanaron, Sabah. 04°42.052'N, 116°36.016'E	AY547381	n.a.
6	*Georissa saulae* (van Benthem Jutting, 1966)	BOR/MOL 2663-2667 (Schilthuizen et al. 2012)	G.saulae_hapC_AY547384_SanaronSepulut Valley, Batu Sanaron, Sabah. 04°42.052'N, 116°36.016'E	AY547384	n.a.
7	*Georissa saulae* (van Benthem Jutting, 1966)	BOR/MOL 3493	G.saulae_ZI003_SanaronSepulut Valley, Batu Sanaron, Sabah. 04°42.052'N, 116°36.016'E	MG982261	n.a.
8	*Georissa saulae* (van Benthem Jutting, 1966)	BOR/MOL 3493	G.saulae_KPH00181.02_SanaronSepulut Valley, Batu Sanaron, Sabah. 04°42.052'N, 116°36.016'E	MG982267	n.a.
9	*Georissa saulae* (van Benthem Jutting, 1966)	BOR/MOL 12770	G.saulae_Sau-001_PungitonSepulut Valley, Gua Pungiton, Sabah. 04°42.410'N, 116°36.040'E	MG982262	n.a.
10	*Georissa saulae* (van Benthem Jutting, 1966)	BOR/MOL 12770	G.saulae_Sau-002_PungitonSepulut Valley, Gua Pungiton, Sabah. 04°42.410'N, 116°36.040'E	MG982263	n.a.
11	*Georissa saulae* (van Benthem Jutting, 1966)	BOR/MOL 12770	G.saulae_Sau-003_PungitonSepulut Valley, Gua Pungiton, Sabah. 04°42.410'N, 116°36.040'E	MG982264	n.a.
12	*Georissa saulae* (van Benthem Jutting, 1966)	BOR/MOL 12770	G.saulae_Sau-004_PungitonSepulut Valley, Gua Pungiton, Sabah. 04°42.410'N, 116°36.040'E	MG982265	n.a.
13	*Georissa saulae* (van Benthem Jutting, 1966)	BOR/MOL 12770	G.saulae_Sau-005_PungitonSepulut Valley, Gua Pungiton, Sabah. 04°42.410'N, 116°36.040'E	MG982266	n.a.
14	*Georissa hosei* Godwin-Austen, 1889	MZU/MOL 16.09	G.hosei_A001_TongakBukit Tongak, Bidi, Bau/Jambusan, Sarawak. 01°22.670'N, 110°08.325'E	MG982327	n.a.
15	*Georissa hosei* Godwin-Austen, 1889	MZU/MOL 16.09	G.hosei_A002_TongakBukit Tongak, Bidi, Bau/Jambusan, Sarawak. 01°22.670'N, 110°08.325'E	MG982331	MH033908
16	*Georissa hosei* Godwin-Austen, 1889	MZU/MOL 16.09	G.hosei_A003_TongakBukit Tongak, Bidi, Bau/Jambusan, Sarawak. 01°22.670'N, 110°08.325'E	MG982330	n.a.
17	*Georissa hosei* Godwin-Austen, 1889	MZU/MOL 16.09	G.hosei_A004_TongakBukit Tongak, Bidi, Bau/Jambusan, Sarawak. 01°22.670'N, 110°08.325'E	MG982329	MH033907
18	*Georissa hosei* Godwin-Austen, 1889	MZU/MOL 16.09	G.hosei_A005_TongakBukit Tongak, Bidi, Bau/Jambusan, Sarawak. 01°22.670'N, 110°08.325'E	MG982328	n.a.
19	*Georissa hosei* Godwin-Austen, 1889	MZU/MOL 16.09	G.hosei_A006_TongakBukit Tongak, Bidi, Bau/Jambusan, Sarawak. 01°22.670'N, 110°08.325'E	MG982326	n.a.
20	*Georissa hosei* Godwin-Austen, 1889	MZU/MOL 16.04	G.hosei_C001_LiakGunung Liak/Padang, Kampung Skiat Baru, Jambusan, Sarawak. 01°24.050'N, 110°11.197'E	MG982339	MH033904
21	*Georissa hosei* Godwin-Austen, 1889	MZU/MOL 16.04	G.hosei_C002_LiakGunung Liak/Padang, Kampung Skiat Baru, Jambusan, Sarawak. 01°24.050'N, 110°11.197'E	MG982338	MH033905
22	*Georissa hosei* Godwin-Austen, 1889	MZU/MOL 16.04	G.hosei_C003_LiakGunung Liak/Padang, Kampung Skiat Baru, Jambusan, Sarawak. 01°24.050'N, 110°11.197'E	MG982341	MH033902
23	*Georissa hosei* Godwin-Austen, 1889	MZU/MOL 16.04	G.hosei_C004_LiakGunung Liak/Padang, Kampung Skiat Baru, Jambusan, Sarawak. 01°24.050'N, 110°11.197'E	MG982340	MH033903
24	*Georissa hosei* Godwin-Austen, 1889	MZU/MOL 16.04	G.hosei_C005_Liak Gunung Liak/Padang, Kampung Skiat Baru, Jambusan, Sarawak. 01°24.050'N, 110°11.197'E	MG982337	n.a.
25	*Georissa hosei* Godwin-Austen, 1889	MZU/MOL 16.04	G.hosei_C006_LiakGunung Liak/Padang, Kampung Skiat Baru, Jambusan, Sarawak. 01°24.050'N, 110°11.197'E	MG982336	MH033906
26	*Georissa hosei* Godwin-Austen, 1889	MZU/MOL 16.04	G.hosei_C007_Liak Gunung Liak/Padang, Kampung Skiat Baru, Jambusan, Sarawak. 01°24.050'N, 110°11.197'E	MG982335	n.a.
27	*Georissa hosei* Godwin-Austen, 1889	MZU/MOL 16.04	G.hosei_C008_Liak Gunung Liak/Padang, Kampung Skiat Baru, Jambusan, Sarawak. 01°24.050'N, 110°11.197'E	MG982334	n.a.
28	*Georissa hosei* Godwin-Austen, 1889	MZU/MOL 16.04	G.hosei_C009_LiakGunung Liak/Padang, Kampung Skiat Baru, Jambusan, Sarawak. 01°24.050'N, 110°11.197'E	MG982333	n.a.
29	*Georissa hosei* Godwin-Austen, 1889	MZU/MOL 16.04	G.hosei_C0010_LiakGunung Liak/Padang, Kampung Skiat Baru, Jambusan, Sarawak. 01°24.050"N, 110°11.197"E	MG982332	n.a.
30	*Georissa hosei* Godwin-Austen, 1889	MZU/MOL 16.08	G.hosei_D001_SiboyuhBukit Siboyuh, Kampung Skiat Baru, Jambusan, Sarawak.01°22.909'N, 110°11.695'E	MG982346	MH033900
31	*Georissa hosei* Godwin-Austen, 1889	MZU/MOL 16.08	G.hosei_D002_SiboyuhBukit Siboyuh, Kampung Skiat Baru, Jambusan, Sarawak.01°22.909'N, 110°11.695'E	MG982342	MH033901
32	*Georissa hosei* Godwin-Austen, 1889	MZU/MOL 16.08	G.hosei_D003_SiboyuhBukit Siboyuh, Kampung Skiat Baru, Jambusan, Sarawak.01°22.909'N, 110°11.695'E	MG982345	MH033898
33	*Georissa hosei* Godwin-Austen, 1889	MZU/MOL 16.08	G.hosei_D004_SiboyuhBukit Siboyuh, Kampung Skiat Baru, Jambusan, Sarawak.01°22.909'N, 110°11.695'E	MG982344	MH033899
34	*Georissa hosei* Godwin-Austen, 1889	MZU/MOL 16.08	G.hosei_D006_SiboyuhBukit Siboyuh, Kampung Skiat Baru, Jambusan, Sarawak.01°22.909'N, 110°11.695'E	MG982343	n.a.
35	*Georissa anyiensis* sp. n.	MZU/MOL 17.50	G.anyiensis_BSP2-01_Bukit SarangPlot 2, Bukit Lebik at Bukit Sarang, Bintulu, Sarawak.02°39.325'N, 113°02.432'E	MG982271	MH033929
36	*Georissa anyiensis* sp. n.	MZU/MOL 17.50	G.anyiensis_BSP2-02_Bukit SarangPlot 2, Bukit Lebik at Bukit Sarang, Bintulu, Sarawak.02°39.325'N, 113°02.432'E	MG982269	MH033930
37	*Georissa anyiensis* sp. n.	MZU/MOL 17.50	G.anyiensis_BSP2-03_Bukit SarangPlot 2, Bukit Lebik at Bukit Sarang, Bintulu, Sarawak.02°39.325'N, 113°02.432'E	MG982268	MH033928
38	*Georissa anyiensis* sp. n.	MZU/MOL 17.50	G.anyiensis_BSP2-04_Bukit SarangPlot 2, Bukit Lebik at Bukit Sarang, Bintulu, Sarawak.02°39.325'N, 113°02.432'E	MG982270	n.a.
39	*Georissa anyiensis* sp. n.	MZU/MOL 17.51	G.anyiensis_BSP11-01_Bukit SarangPlot 11, Bukit Lebik at Bukit Sarang, Bintulu, Sarawak.02°39.325'N, 113°02.432'E	n.a.	MH033926
40	*Georissa anyiensis* sp. n.	MZU/MOL 17.51	G.anyiensis_BSP11-02_Bukit SarangPlot 11, Bukit Lebik at Bukit Sarang, Bintulu, Sarawak.02°39.325'N, 113°02.432'E	MG982278	MH033927
41	*Georissa anyiensis* sp. n.	MZU/MOL 17.51	G.anyiensis_BSP11-03_Bukit SarangPlot 11, Bukit Lebik at Bukit Sarang, Bintulu, Sarawak.02°39.325'N, 113°02.432'E	MG982280	MH033924
42	*Georissa anyiensis* sp. n.	MZU/MOL 17.51	G.anyiensis_BSP11-04_Bukit SarangPlot 11, Bukit Lebik at Bukit Sarang, Bintulu, Sarawak.02°39.325'N, 113°02.432'E	MG982279	MH033925
43	*Georissa anyiensis* sp. n.	MZU/MOL 17.60	G.anyiensis_BSP22-01_Bukit SarangPlot 22, Bukit Anyi at Bukit Sarang, Bintulu, Sarawak.02°39.252'N, 113°02.723'E	MG982272	n.a.
44	*Georissa anyiensis* sp. n.	MZU/MOL 17.60	G.anyiensis_BSP22-02_Bukit SarangPlot 22, Bukit Anyi at Bukit Sarang, Bintulu, Sarawak.02°39.252'N, 113°02.723'E	MG982273	MH033931
45	*Georissa anyiensis* sp. n.	MZU/MOL 17.60	G.anyiensis_BSP22-03_Bukit SarangPlot 22, Bukit Anyi at Bukit Sarang, Bintulu, Sarawak.02°39.252'N, 113°02.723'E	MG982274	MH033933
46	*Georissa anyiensis* sp. n.	MZU/MOL 17.60	G.anyiensis_BSP22-04_Bukit SarangPlot 22, Bukit Anyi at Bukit Sarang, Bintulu, Sarawak.02°39.252'N, 113°02.723'E	MG982275	MH033934
47	*Georissa anyiensis* sp. n.	MZU/MOL 17.60	G.anyiensis_BSP22-05_Bukit SarangPlot 22, Bukit Anyi at Bukit Sarang, Bintulu, Sarawak.02°39.252'N, 113°02.723'E	MG982276	MH033935
48	*Georissa anyiensis* sp. n.	MZU/MOL 17.60	G.anyiensis_BSP22-06_Bukit SarangPlot 22, Bukit Anyi at Bukit Sarang, Bintulu, Sarawak.02°39.252'N, 113°02.723'E	MG982277	MH033932
49	*Georissa muluensis* sp. n.	MZU/MOL 17.31	G.muluensis_LGG-01_MuluPlot 1, Lagang Cave, Mulu National Park, Mulu, Sarawak.04°03.060'N, 114°49.372'E	MG982288	MH033893
50	*Georissa muluensis* sp. n.	MZU/MOL 17.31	G.muluensis_LGG-02_MuluPlot 1, Lagang Cave, Mulu National Park, Mulu, Sarawak.04°03.060'N, 114°49.372'E	MG982285	MH033891
51	*Georissa muluensis* sp. n.	MZU/MOL 17.31	G.muluensis_LGG-03_MuluPlot 1, Lagang Cave, Mulu National Park, Mulu, Sarawak.04°03.060'N, 114°49.372'E	MG982286	MH033892
52	*Georissa muluensis* sp. n.	MZU/MOL 17.31	G.muluensis_LGG-04_MuluPlot 1, Lagang Cave, Mulu National Park, Mulu, Sarawak.04°03.060'N, 114°49.372'E	MG982287	MH033890
53	*Georissa hadra* Thompson & Dance, 1983	MZU/MOL 17.32	G.hadra_LC-01_MuluLang Cave, Mulu N.P., Mulu, Sarawak. 04°01.490'N, 114°49.482'E	MG982284	MH033896
54	*Georissa hadra* Thompson & Dance, 1983	MZU/MOL 17.32	G.hadra_LC-02_MuluLang Cave, Mulu N.P., Mulu, Sarawak. 04°01.490'N, 114°49.482'E	MG982282	MH033897
55	*Georissa hadra* Thompson & Dance, 1983	MZU/MOL 17.32	G.hadra_LC-03_MuluLang Cave, Mulu N.P., Mulu, Sarawak. 04°01.490'N, 114°49.482'E	MG982281	MH033894
56	*Georissa hadra* Thompson & Dance, 1983	MZU/MOL 17.32	G.hadra_LC-04_MuluLang Cave, Mulu N.P., Mulu, Sarawak. 04°01.490'N, 114°49.482'E	MG982283	MH033895
57	*Georissa kobelti* Gredler, 1902	MZU/MOL 17.36	G.kobelti_TC-01_NiahTrade Cave, Niah National Park, Niah, Sarawak. 03°49.137'N, 113°46.860'E	MG982296	MH033886
58	*Georissa kobelti* Gredler, 1902	MZU/MOL 17.36	G.kobelti_TC-02_NiahTrade Cave, Niah National Park, Niah, Sarawak.03°49.137'N, 113°46.860'E	MG982295	MH033889
59	*Georissa kobelti* Gredler, 1902	MZU/MOL 17.36	G.kobelti_TC-03_NiahTrade Cave, Niah National Park, Niah, Sarawak.03°49.137'N, 113°46.860'E	MG982293	MH033887
60	*Georissa kobelti* Gredler, 1902	MZU/MOL 17.36	G.kobelti_TC-04_NiahTrade Cave, Niah National Park, Niah, Sarawak.03°49.137'N, 113°46.860'E	MG982294	MH033888
61	*Georissa kobelti* Gredler, 1902	MZU/MOL 17.38	G.kobelti_KJ1-01_BaramPlot 1, Bukit Kaijin, Baram, Sarawak. 03°41.753'N, 114°27.555'E	MG982290	MH033882
62	*Georissa kobelti* Gredler, 1902	MZU/MOL 17.38	G.kobelti_KJ1-02_BaramPlot 1, Bukit Kaijin, Baram, Sarawak. 03°41.753'N, 114°27.555'E	MG982289	MH033883
63	*Georissa kobelti* Gredler, 1902	MZU/MOL 17.38	G.kobelti_KJ1-03_BaramPlot 1, Bukit Kaijin, Baram, Sarawak. 03°41.753'N, 114°27.555'E	MG982292	MH033885
64	*Georissa kobelti* Gredler, 1902	MZU/MOL 17.38	G.kobelti_KJ1-04_BaramPlot 1, Bukit Kaijin, Baram, Sarawak. 03°41.753'N, 114°27.555'E	MG982291	MH033884
65	*Georissa niahensis* Godwin-Austen, 1889	MZU/MOL 17.25	G.niahensis_PC-01_NiahPainted Cave, Niah National Park, Niah, Sarawak.03°48.688'N, 113°47.250'E	MG982301	MH033965
66	*Georissa niahensis* Godwin-Austen, 1889	MZU/MOL 17.25	G.niahensis_PC-02_NiahPainted Cave, Niah National Park, Niah, Sarawak.03°48.688'N, 113°47.250'E	MG982300	MH033878
67	*Georissa niahensis* Godwin-Austen, 1889	MZU/MOL 17.25	G.niahensis_PC-03_NiahPainted Cave, Niah National Park, Niah, Sarawak.03°48.688'N, 113°47.250'E	MG982297	MH033877
68	*Georissa niahensis* Godwin-Austen, 1889	MZU/MOL 17.25	G.niahensis_PC-04_NiahPainted Cave, Niah National Park, Niah, Sarawak.03°48.688'N, 113°47.250'E	MG982298	MH033954
69	*Georissa niahensis* Godwin-Austen, 1889	MZU/MOL 17.25	G.niahensis_GC-01_NiahPainted Cave, Niah National Park, Niah, Sarawak.03°48.688'N, 113°47.250'E	MG982299	MH033879
70	*Georissa niahensis* Godwin-Austen, 1889	MZU/MOL 17.25	G.niahensis_GC-02_NiahPainted Cave, Niah National Park, Niah, Sarawak.03°48.688'N, 113°47.250'E	MG982302	MH033880
71	*Georissa niahensis* Godwin-Austen, 1889	MZU/MOL 17.25	G.niahensis_GC-03_NiahPainted Cave, Niah National Park, Niah, Sarawak.03°48.688'N, 113°47.250'E	MG982304	n.a.
72	*Georissa niahensis* Godwin-Austen, 1889	MZU/MOL 17.25	G.niahensis_GC-04_NiahPainted Cave, Niah National Park, Niah, Sarawak.03°48.688'N, 113°47.250'E	MG982303	MH033881
73	*Georissa silaburensis* sp. n.	MZU/MOL 17.05	G.silaburensis_SIG3-01_SilaburPlot 3, Gunong Silabur, Serian, Sarawak. 00°57.285'N, 110°30.228'E	MG982323	MH033949
74	*Georissa silaburensis* sp. n.	MZU/MOL 17.05	G.silaburensis_SIG3-03_SilaburPlot 3, Gunong Silabur, Serian, Sarawak. 00°57.285'N, 110°30.228'E	MG982324	MH033948
75	*Georissa silaburensis* sp. n.	MZU/MOL 17.05	G.silaburensis_SIG3-05_SilaburPlot 3, Gunong Silabur, Serian, Sarawak. 00°57.285'N, 110°30.228'E	MG982325	MH033944
76	*Georissa silaburensis* sp. n.	MZU/MOL 17.06	G.silaburensis_SIG4-01_SilaburPlot 4, Gunong Silabur, Serian, Sarawak. 00°57.285'N, 110°30.228'E	MG982320	MH033945
77	*Georissa silaburensis* sp. n.	MZU/MOL 17.06	G.silaburensis_SIG4-03_SilaburPlot 4, Gunong Silabur, Serian, Sarawak. 00°57.285'N, 110°30.228'E	MG982321	MH033952
78	*Georissa silaburensis* sp. n.	MZU/MOL 17.06	G.silaburensis_SIG4-06_SilaburPlot 4, Gunong Silabur, Serian, Sarawak. 00°57.285'N, 110°30.228'E	MG982322	MH033951
79	*Georissa silaburensis* sp. n.	MZU/MOL 17.07	G.silaburensis_SIG5-07_SilaburPlot 5, Gunong Silabur, Serian, Sarawak. 00°57.285'N, 110°30.228'E	MG982316	MH033946
80	*Georissa silaburensis* sp. n.	MZU/MOL 17.07	G.silaburensis_SIG5-08_SilaburPlot 5, Gunong Silabur, Serian, Sarawak. 00°57.285'N, 110°30.228'E	MG982317	MH033950
81	*Georissa silaburensis* sp. n.	MZU/MOL 17.07	G.silaburensis_SIG5-09_SilaburPlot 5, Gunong Silabur, Serian, Sarawak. 00°57.285'N, 110°30.228'E	MG982318	n.a.
82	*Georissa silaburensis* sp. n.	MZU/MOL 17.07	G.silaburensis_SIG5-10_SilaburPlot 5, Gunong Silabur, Serian, Sarawak. 00°57.285'N, 110°30.228'E	MG982319	MH033947
83	*Georissa bauensis* sp. n.	MZU/MOL 16.01	G.bauensis_B002_WCaveWind Cave Passage 3, Wind Cave National Park, Bau, Sarawak. 01°24.810'N, 110°08.175'E	MG982306	MH033937
84	*Georissa bauensis* sp. n.	MZU/MOL 16.01	G.bauensis_B003_WCaveWind Cave Passage 3, Wind Cave National Park, Bau, Sarawak. 01°24.810'N, 110°08.175'E	n.a.	MH033938
85	*Georissa bauensis* sp. n.	MZU/MOL 16.01	G.bauensis_B004_WCaveWind Cave Passage 3, Wind Cave National Park, Bau, Sarawak. 01°24.810'N, 110°08.175'E	MG982309	MH033936
86	*Georissa bauensis* sp. n.	MZU/MOL 16.01	G.bauensis_B005_WCaveWind Cave Passage 3, Wind Cave National Park, Bau, Sarawak. 01°24.810'N, 110°08.175'E	MG982307	n.a.
87	*Georissa bauensis* sp. n.	MZU/MOL 16.01	G.bauensis_B007_WCaveWind Cave Passage 3, Wind Cave National Park, Bau, Sarawak. 01°24.810'N, 110°08.175'E	MG982308	n.a.
88	*Georissa bauensis* sp. n.	MZU/MOL 16.01	G.bauensis_B008_WCaveWind Cave Passage 3, Wind Cave National Park, Bau, Sarawak. 01°24.810'N, 110°08.175'E	MG982311	MH033939
89	*Georissa bauensis* sp. n.	MZU/MOL 16.01	G.bauensis_B009_WCaveWind Cave Passage 3, Wind Cave National Park, Bau, Sarawak.01°24.810'N, 110°08.175'E	MG982305	n.a.
90	*Georissa bauensis* sp. n.	MZU/MOL 16.01	G.bauensis_B010_WCaveWind Cave Passage 3, Wind Cave National Park, Bau, Sarawak.01°24.810'N, 110°08.175'E	MG982310	n.a.
91	*Georissa bauensis* sp. n.	MZU/MOL 16.03	G.bauensis_Q001_AyubGunong Podam, near Sg. Ayup, Kampung Bogag, Bau, Sarawak.01°21.158'N, 110°03.577'E	MG982313	MH033942
92	*Georissa bauensis* sp. n.	MZU/MOL 16.03	G.bauensis_Q002_AyubGunong Podam, near Sg. Ayup, Kampung Bogag, Bau, Sarawak.01°21.158'N, 110°03.577'E	MG982312	n.a.
93	*Georissa bauensis* sp. n.	MZU/MOL 16.03	G.bauensis_Q003_AyubGunong Podam, near Sg. Ayup, Kampung Bogag, Bau, Sarawak.01°21.158'N, 110°03.577'E	MG982314	n.a.
94	*Georissa bauensis* sp. n.	MZU/MOL 16.03	G.bauensis_Q004_AyubGunong Podam, near Sg. Ayup, Kampung Bogag, Bau, Sarawak.01°21.158'N, 110°03.577'E	n.a.	MH033941
95	*Georissa bauensis* sp. n.	MZU/MOL 16.03	G.bauensis_Q005_AyubGunong Podam, near Sg. Ayup, Kampung Bogag, Bau, Sarawak.01°21.158'N, 110°03.577'E	n.a.	MH033940
96	*Georissa bauensis* sp. n.	MZU/MOL 16.03	G.bauensis_Q006_AyubGunong Podam, near Sg. Ayup, Kampung Bogag, Bau, Sarawak.01°21.158'N, 110°03.577'E	MG982315	MH033943
97	*Georissa pyrrhoderma* Thompson & Dance, 1983	MZU/MOL 17.11	G.pyrrhoderma_SO3-01_SilaburPlot Outside 3-1, Gunong Silabur, Serian, Sarawak.00°57.451'N, 110°30.207'E	MG982366	MH033913
98	*Georissa pyrrhoderma* Thompson & Dance, 1983	MZU/MOL 17.11	G.pyrrhoderma_SO3-02_SilaburPlot Outside 3-1, Gunong Silabur, Serian, Sarawak.00°57.451'N, 110°30.207'E	MG982364	MH033914
99	*Georissa pyrrhoderma* Thompson & Dance, 1983	MZU/MOL 17.11	G.pyrrhoderma_SO3-03_SilaburPlot Outside 3-1, Gunong Silabur, Serian, Sarawak.00°57.451'N, 110°30.207'E	MG982367	MH033915
100	*Georissa pyrrhoderma* Thompson & Dance, 1983	MZU/MOL 17.11	G.pyrrhoderma_SO3-04_SilaburPlot Outside 3-1, Gunong Silabur, Serian, Sarawak.00°57.451'N, 110°30.207'E	MG982365	MH033916
101	*Georissa pyrrhoderma* Thompson & Dance, 1983	MZU/MOL 17.22	G.pyrrhoderma_SIO4-01_SilaburPlot SIO4, Gunong Silabur, Serian, Sarawak. 00°57.451'N, 110°30.207'E	MG982376	MH033918
102	*Georissa pyrrhoderma* Thompson & Dance, 1983	MZU/MOL 17.22	G.pyrrhoderma_SIO4-02_SilaburPlot SIO4, Gunong Silabur, Serian, Sarawak. 00°57.451'N, 110°30.207'E	MG982377	MH033920
103	*Georissa pyrrhoderma* Thompson & Dance, 1983	MZU/MOL 17.22	G.pyrrhoderma_SIO4-03_SilaburPlot SIO4, Gunong Silabur, Serian, Sarawak. 00°57.451'N, 110°30.207'E	MG982378	MH033917
104	*Georissa pyrrhoderma* Thompson & Dance, 1983	MZU/MOL 17.22	G.pyrrhoderma_SIO4-04_SilaburPlot SIO4, Gunong Silabur, Serian, Sarawak. 00°57.451'N, 110°30.207'E	MG982379	MH033919
105	*Georissa pyrrhoderma* Thompson & Dance, 1983	MZU/MOL 17.22	G.pyrrhoderma_SIO4-05_SilaburPlot SIO4, Gunong Silabur, Serian, Sarawak. 00°57.451'N, 110°30.207'E	MG982380	n.a.
106	*Georissa pyrrhoderma* Thompson & Dance, 1983	MZU/MOL 17.13	G.pyrrhoderma_SIE1-01_SilaburPlot SIE1, Gunong Silabur, Serian, Sarawak. 00°57.451'N, 110°30.207'E	MG982372	n.a.
107	*Georissa pyrrhoderma* Thompson & Dance, 1983	MZU/MOL 17.13	G.pyrrhoderma_SIE1-02_SilaburPlot SIE1, Gunong Silabur, Serian, Sarawak. 00°57.451'N, 110°30.207'E	MG982373	MH033922
108	*Georissa pyrrhoderma* Thompson & Dance, 1983	MZU/MOL 17.13	G.pyrrhoderma_SIE1-03_SilaburPlot SIE1, Gunong Silabur, Serian, Sarawak. 00°57.451'N, 110°30.207'E	MG982374	MH033923
109	*Georissa pyrrhoderma* Thompson & Dance, 1983	MZU/MOL 17.13	G.pyrrhoderma_SIE1-04_SilaburPlot SIE1, Gunong Silabur, Serian, Sarawak. 00°57.451'N, 110°30.207'E	MG982375	MH033921
110	*Georissa pyrrhoderma* Thompson & Dance, 1983	MZU/MOL 17.16	G.pyrrhoderma_SIE4-01_SilaburPlot SIE4, Gunong Silabur, Serian, Sarawak. 00°57.451'N, 110°30.207'E	MG982368	MH033910
111	*Georissa pyrrhoderma* Thompson & Dance, 1983	MZU/MOL 17.16	G.pyrrhoderma_SIE4-02_SilaburPlot SIE4, Gunong Silabur, Serian, Sarawak. 00°57.451'N, 110°30.207'E	MG982369	MH033909
112	*Georissa pyrrhoderma* Thompson & Dance, 1983	MZU/MOL 17.16	G.pyrrhoderma_SIE4-03_SilaburPlot SIE4, Gunong Silabur, Serian, Sarawak. 00°57.451'N, 110°30.207'E	MG982370	MH033911
113	*Georissa pyrrhoderma* Thompson & Dance, 1983	MZU/MOL 17.16	G.pyrrhoderma_SIE4-04_SilaburPlot SIE4, Gunong Silabur, Serian, Sarawak. 00°57.451'N, 110°30.207'E	MG982371	MH033912
114	*Georissa kinabatanganensis* sp. n.	BOR/MOL 7289	G.kinabatanganensis_KPH01720.01_PangiKinabatangan Valley, Pangi, Sabah. 05°32.291'N, 118°18.376'E	MG982348	MH033963
115	*Georissa kinabatanganensis* sp. n.	BOR/MOL 7289	G.kinabatanganensis_KPH01720.02_PangiKinabatangan Valley, Pangi, Sabah. 05°32.291'N, 118°18.376'E	MG982347	MH033962
116	*Georissa kinabatanganensis* sp. n.	BOR/MOL 7289	G.kinabatanganensis_KPH01720.03_PangiKinabatangan Valley, Pangi, Sabah. 05°32.291'N, 118°18.376'E	n.a.	MH033961
117	*Georissa kinabatanganensis* sp. n.	MZU/MOL 17.26	G.kinabatanganensis_K001_KeruakKeruak, near Kinabatangan river, Sandakan, Sabah. 05°32.291'N, 118°18.376'E	MG982349	MH033959
118	*Georissa kinabatanganensis* sp. n.	MZU/MOL 17.26	G.kinabatanganensis_K002_KeruakKeruak, near Kinabatangan river, Sandakan, Sabah.05°31.385'N, 118°17.113'E	MG982351	MH033958
119	*Georissa kinabatanganensis* sp. n.	MZU/MOL 17.26	G.kinabatanganensis_K005_KeruakKeruak, near Kinabatangan river, Sandakan, Sabah.05°31.385'N, 118°17.113'E	MG982350	MH033960
120	*Georissa sepulutensis* sp. n.	BOR/MOL 39	G.sepulutensis_KPH00176.01_SanaronSepulut Valley, Batu Sanaron, Sabah. 04°42.052'N, 116°36.016'E	MG982357	MH033957
121	*Georissa sepulutensis* sp. n.	BOR/MOL 39	G.sepulutensis_KPH00176.02_SanaronSepulut Valley, Batu Sanaron, Sabah. 04°42.052'N, 116°36.016'E	MG982356	n.a.
122	*Georissa sepulutensis* sp. n.	BOR/MOL 39	G.sepulutensis_KPH00181.01_SanaronSepulut Valley, Batu Sanaron, Sabah. 04°42.052'N, 116°36.016'E	MG982359	MH033956
123	*Georissa sepulutensis* sp. n.	BOR/MOL 12278	G.sepulutensis_Sca-002_PungitonSepulut Valley, Gua Pungiton, Sabah. 04°42.410'N, 116°36.040'E	MG982361	MH033964
124	*Georissa sepulutensis* sp. n.	BOR/MOL 12278	G.sepulutensis_Sca-003_PungitonSepulut Valley, Gua Pungiton, Sabah. 04°42.410'N, 116°36.040'E	MG982360	MH033955
125	*Georissa sepulutensis* sp. n.	BOR/MOL 12278	G.sepulutensis_Sca-004_PungitonSepulut Valley, Gua Pungiton, Sabah. 04°42.410'N, 116°36.040'E	MG982362	MH033953
126	*Georissa sepulutensis* sp. n.	BOR/MOL 12278	G.sepulutensis_Sca-005_PungitonSepulut Valley, Gua Pungiton, Sabah. 04°42.410'N, 116°36.040'E	MG982363	n.a.
127	*Georissa sepulutensis* sp. n.	BOR/MOL 39	G.sepulutensis_ZA004_SanaronSepulut Valley, Batu Sanaron, Sabah. 04°42.052'N, 116°36.016'E	MG982354	n.a.
128	*Georissa sepulutensis* sp. n.	BOR/MOL 39	G.sepulutensis_ZB003_SanaronSepulut Valley, Batu Sanaron, Sabah. 04°42.052'N, 116°36.016'E	MG982355	n.a.
129	*Georissa sepulutensis* sp. n.	BOR/MOL 39	G.sepulutensis_ZC003_SanaronSepulut Valley, Batu Sanaron, Sabah. 04°42.052'N, 116°36.016'E	MG982358	n.a.
130	*Georissa sepulutensis* sp. n.	RMNH/MOL 333905	G.sepulutensis_ZE003_SimbaluyonSepulut Valley, Bukit Simbaluyon, Sabah. 04°43.200'N, 116°34.140'E	MG982352	n.a.
131	*Georissa sepulutensis* sp. n.	RMNH/MOL 333905	G.sepulutensis_ZE004_SimbaluyonSepulut Valley, Bukit Simbaluyon, Sabah. 04°43.200'N, 116°34.140'E	MG982353	n.a.
132	*Georissa sepulutensis* sp. n.	BOR/MOL 39 (Schilthuizen et al., 2005)	G.sepulutensis_hapA_AY547387_SanaronSepulut Valley, Batu Sanaron, Sabah. 04°42.052'N, 116°36.016'E	AY547387	n.a.
133	*Georissa sepulutensis* sp. n.	BOR/MOL 39 (Schilthuizen et al., 2005)	G.sepulutensis_hapB_AY547388_SanaronSepulut Valley, Batu Sanaron, Sabah. 04°42.052'N, 116°36.016'E	AY547388	n.a.

### Sequence alignment and phylogenetic analyses

**Sequence data and alignement**. A total of 12 ingroup taxa of “scaly group” *Georissa* including an outgroup taxon, *Georissa gomantongensis* Smith, 1893, were used for phylogenetic analyses (using a much larger hydrocenid taxon sampling, to be published elsewhere, we confirmed that *G. gomantongensis* indeed branches off basally to the “scaly group”). We added another six 16S mtDNA sequences from GenBank, representing *Georissa saulae* (van Benthem-Jutting, 1966) (GenBank accession no. AY547380, AY547381, AY547384, and AY547385) and *Georissa sepulutensis* sp. n. (GenBank accession no. AY547387 and AY548388). We conducted our phylogenetic analyses based on 128 sequences for 16S and 91 sequences for CO1. The forward and reverse nucleotide reads were assembled using *de novo* Geneious 10.0.7 assembler, manually checked and edited, and later aligned using default settings of MUSCLE alignment (Edgar 2004). **Phylogenetic inference**. For CO1 sequences, we selected the invertebrate mitochondrial genetic code at the second reading frame. Ambiguous nucleotide sequence ends were trimmed and removed prior to further analysis. ModelFinder (Kalyaanamoorthy et al. 2017) was used to select the most appropriate model, based on the corrected Akaike Information Creterion (AICc) for partial 16S and CO1 mtDNA genes. The best fitting models were TIM3+F+I+G4 for 16S and TIM2+F+I+G4 for CO1. **Phylogenetic analysis**. Maximum likelihood analysis was performed using IQ-TREE 1.6.3 (Nguyen et al. 2014) on a concatenated 16S and CO1 sequences of “scaly” *Georissa* using TIM3+F+I+G4 as the nucleotide substitution models with ultrafast bootstrapping (1000 replicates) (Hoang et al. 2017). Bayesian Inference was performed using MrBayes 3.2.6 (Huelsenbeck and Ronquist 2001) with the next closest nucleotide substitution model, GTR+I+G using the following MCMC settings: Chain length = 1,100,000 generations, heated chain = 4, subsampling frequency = one tree for each 200 generations, burn-in length = 100,000, and chain temperature = 0.2.

### Species delimitation and description

For species delimitation, we combined the data of molecular phylogenetic analyses and the assessments of the morphology. We aimed for monophyly in species, allowing paraphyly under certain circumstances (Schilthuizen and Gittenberger 1996), but disallowing polyphyly. Only when we found morphological characters that could distinguish DNA-based clades or paraphyletic groups, did we consider such groups as potential species. Although many forms in *Georissa* are allopatric, we did have a number of cases where two forms occurred sympatrically without forming intermediates, which also aided in determining species status by application of the biological species concept (Mayr 1942). General shell characters were further divided into detailed sub-characters exclusively for the descriptions of the representatives of the “scaly group” of Bornean *Georissa*. The assessed morphological characters follow the descriptions made by Godwin-Austen (1889), Gredler (1902), Haase and Schilthuizen (2007), Smith (1893, 1895), Thompson and Dance (1983), van Benthem-Jutting (1966), Vermeulen and Junau (2007), and Vermeulen et al. (2015). Note that color indications always refer to living or freshly dead specimens, as the color in older specimens usually degrades, with an exception for *Georissa scalinella* (van Benthem-Jutting, 1966), where only old collection specimens were available.

### CO1 genetic divergence

In addition to the molecular phylogenetic and morphological assessment in our species delimitation, we conducted divergence analysis of partial CO1 genes to provide additional information to assist in the species delimitation of “scaly” *Georissa*. Several other studies on species delimitation in gastropods have also used CO1 mtDNA successfully (see Liew et al. 2014, Puillandre et al. 2012a, 2012b). Pairwise genetic distances of CO1 sequences from 89 individuals were computed based on Kimura 2-parameter with MEGA v. 7.0.26 (Kumar et al. 2016). These comprised of eleven species, including the six new species.

### Web interface species delimitation using 16S mtDNA

We carried out two more approaches of web interface species delimitation to provide more insight in our species delimitation, namely, Automatic Barcode Gap Discovery (ABGD) (http://wwwabi.snv.jussieu.fr/public/abgd/abgdweb.html) (Puillandre et al. 2012a), and Poisson Tree Processes (PTP) (http://species.h-its.org/ptp/) (Zhang et al. 2013). ABGD analysis was carried out using 16S mtDNA sequences of the “scaly group” *Georissa* (excluding the outgroup). The parameters were set to default. For PTP analysis, we used the 16S gene tree generated from IQ-TREE (Nguyen et al. 2014). The parameters were set to default. Both ABGD and PTP analyses were conducted using mtDNA 16S sequences and gene tree based on the available data of all studied taxa. ABGD aims to partition the species based on the barcode gap (Puillandre et al. 2012a), while PTP focuses on the branching event of a rooted phylogenetic tree (Zhang et al. 2013).

## Results and discussion

### Morphology and phylogenetic analyses

**Figure 1.**
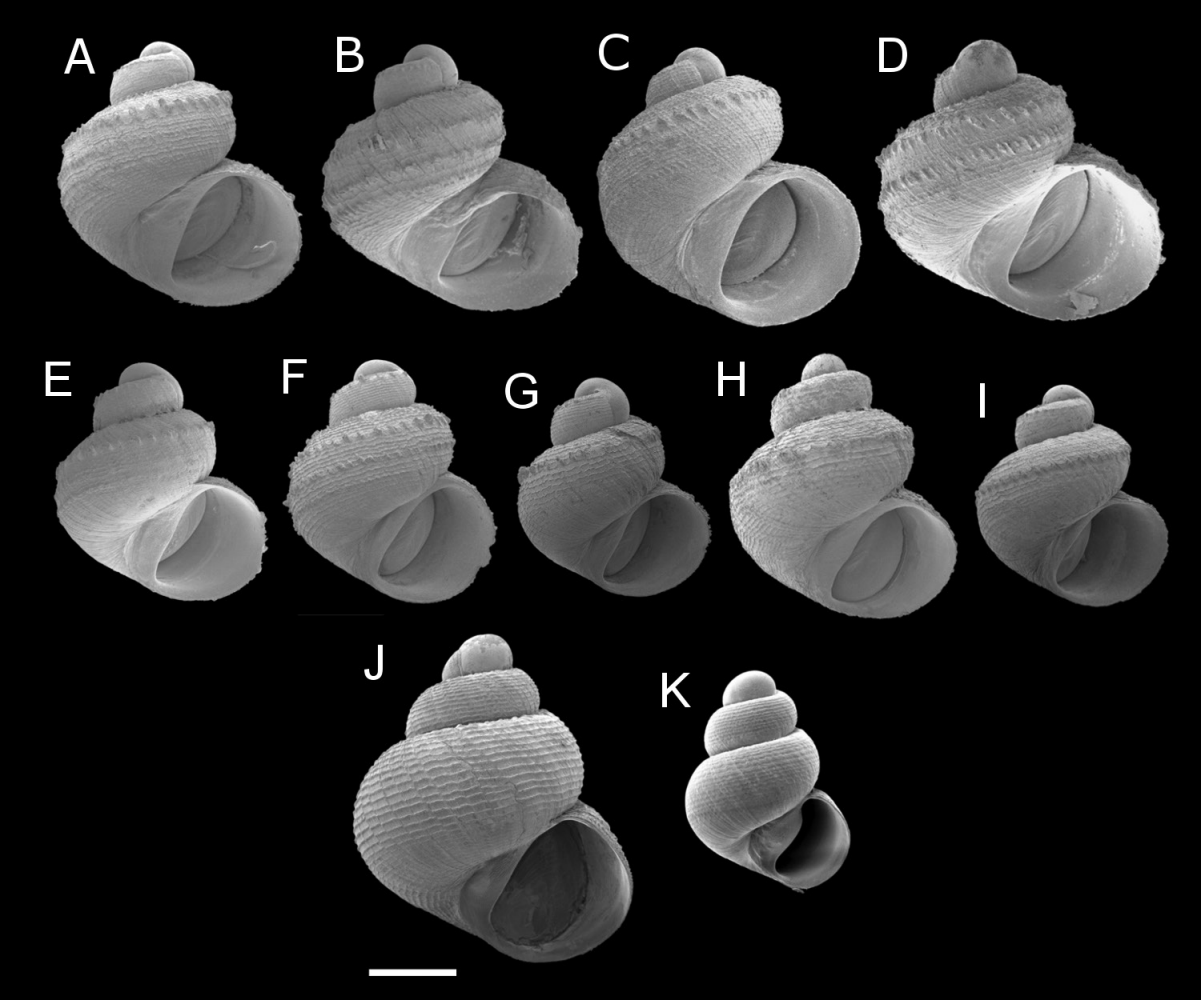
**A–D** Intraspecific variation in shell shape and sculpture of *Georissa hosei* Godwin-Austen, 1889 **E–G** Intraspecific variation in shell shape and sculpture of *Georissa bauensis* sp. n. **H** *Georissa pyrrhoderma* Thompson & Dance, 1983 **I** *Georissa sepulutensis* sp. n. For comparison with the “scaly group”, two additional species are shown that do not belong to the “scaly group”, namely: **J** *Georissa gomantongensis* Smith, 1893 and **K** *Georissa nephrostoma* Vermeulen et al., 2015. Localities: **A, B** Gunung Liak/Padang (Jambusan, Sarawak) **C** Bukit Siboyuh (Jambusan, Sarawak) **D** Bukit Tongak (Bau/Jambusan, Sarawak) **E, F** Gunung Podam (Bau, Sarawak) **G** Wind Cave Nature Reserve (Bau, Sarawak) **H** Gunung Silabur (Serian, Sarawak) **I** Batu Sanaron (Sanaron, Sabah) **J** Gua Gomantong (Gomantong, Sabah) **K** Keruak (Kinabatangan, Sabah). Scale bar 500 µm.

Our morphological and phylogenetic studies lead us to conclude that there are at least 13 species of “scaly group” *Georissa* currently existing in Malaysian Borneo (for detailed morphological species descriptions, see the species treatments under the Taxonomy section). For one of these, *Georissa scalinella* (van Benthem-Jutting, 1966), DNA data are yet unavailable. Detailed conchological assessments of the “scaly group” show that at least two species, *Georissa bauensis* sp. n. and *Georissa hosei* Godwin-Austen, 1889, are highly variable (both intra- and inter-populationally) with regard to the “scaly” shell microsculpture characters (see Fig. 1). Due to the high inter- and intraspecific variation of these species, identification based on morphological characters alone could be problematic without prior knowledge of the shell variation within these species. Furthermore, species similar in shell habitus and scale characters, like *Georissa pyrrhoderma* Thompson & Dance, 1983 and *Georissa sepulutensis* sp. n., often have character combinations that overlap with either *G. bauensis* or *G. hosei*. Therefore, for identification of “scaly group” specimens, we found thorough conchological examination of the shells aided with molecular data is most reliable.

Based on the molecular phylogenetic analyses of the “scaly group” *Georissa* we find multiple strongly supported monophyletic groups (bootstrap and posterior output values ranging from 89–100 and 97–100, respectively) which correspond with subtly different conchologies. In contrast, *Georissa kobelti* Gredler, 1902 is paraphyletic, and we treat this as a single species based on the conchological characters that support they are conspecific.

### CO1 genetic divergence

Despite geographic proximity for some populations of morphologically highly similar forms, the CO1 divergence analysis shows high genetic divergences (e.g. *G. bauensis* vs. *G. hosei*, genetic divergence = 0.12). For some other species, the interspecific genetic divergence is lower, but such species may be surprisingly distinct in shell sculpture (e.g. *G. hadra* vs. *G. muluensis*, genetic divergence = 0.07). As a consequence, we have sometimes given priority to genetic distinctness, sometimes to morphological distinctness in delimiting species, which means that intraspecific diversity may vary between species. For example, we found that *G. pyrrhoderma*, *G. hosei*, and *G. kobelti* are the three species to have the highest intraspecific divergence (0.06, 0.06 and 0.07, respectively) compared with the rest of the “scaly group”, where all other species have an intraspecific divergence equal to or lower than 0.05 (see details in Table 2). Our study reveals that within group divergences of “scaly” *Georissa* does not exceed 0.07 for each species, while the divergences between all species pairs exceed 0.10, with the exception of *G. kinabatanganensis* vs. *G. sepulutensis*, *G. bauensis* vs. *G. silaburensis*, *G. hadra* vs. *G. muluensis* and *G. kobelti* vs. *G. niahensis*.

**Table 2.** Intra- and inter-specific CO1 sequence divergence of eleven species of “scaly” *Georissa*.

		Divergence within group	Number of specimens	*G. kinabatanganensis*	*G. sepulutensis*	*G. bauensis*	*G. silaburensis*	*G. anyiensis*	*G. pyrrhoderma*	*G. hosei*	*G. hadra*	*G. muluensis*	*G. kobelti*	*G. niahensis*
1	*G. kinabatanganensis*	0.05	6											
2	*G. sepulutensis*	0.02	5	0.06*										
3	*G. bauensis*	0.03	8	0.11	0.14									
4	*G. silaburensis*	<0.01	9	0.12	0.13	0.04*								
5	*G. anyiensis*	0.04	12	0.14	0.14	0.12	0.12							
6	*G. pyrrhoderma*	0.06	15	0.10	0.11	0.11	0.11	0.09						
7	*G. hosei*	0.06	11	0.14	0.13	0.12	0.12	0.10	0.12					
8	*G. hadra*	<0.01	4	0.18	0.18	0.16	0.15	0.12	0.15	0.14				
9	*G. muluensis*	<0.01	4	0.17	0.19	0.15	0.15	0.14	0.14	0.14	0.07*			
10	*G. kobelti*	0.07	8	0.11	0.13	0.12	0.12	0.09	0.10	0.09	0.10	0.09		
11	*G. niahensis*	0.04	7	0.13	0.15	0.13	0.14	0.10	0.12	0.10	0.11	0.13	0.05*	

*The average number of net base substitutions per site between species is equal or lower than 0.07, which is the highest number of base substitution per site within a “scaly” species.

### Web interface species delimitation using 16S mtDNA

To test to what extent automated procedures, based on genetic data alone, could reproduce our subjective species delimitation, we carried out ABGD and PTP analyses. ABGD recursive partition divided the “scaly group” *Georissa* into no more than six species at the lowest intraspecific divergence, while the highest divergence grouped all “scaly group” *Georissa* into a single species. The ABGD analysis further showed that partitioning into six species was due mostly to the separation of *G. saulae* into five different species while the rest of “scaly” *Georissa* were considered as a single species. This is possible due to the even higher intraspecific divergence of 16S mtDNA of *G. saulae* compared to the rest of “scaly group” taxa (see Suppl. material 2).

While ABGD analysis underestimated the number of possible species in the “scaly group” of *Georissa*, PTP analysis based on maximum likelihood delimitation results devided the taxa in at least 15 possible species. The results from this species delimitation method therefore more closely match our preferred approach (in which we combined phylogenetic and morphometric assessment). The PTP analysis does, however, differ from our preferred delimitation at several crucial points. *G. saulae*, *G. kinabatanganensis*, *G. hosei*, *G. kobelti*, and *G. niahensis* are each split into two species, whereas the two sets of species composed of (i) *G. hadra* and *G. muluensis*, and (ii) *G. bauensis* and *G. silaburensis*, are each considered as a single species, which make another two species. Otherwise, PTP analysis resolves the same species as in our preferred resolution (see Suppl. material 3).

The results from CO1 barcoding, ABGD, and PTP analyses reveal that objective species delimitation based solely on molecular data will not be successful for “scaly group” *Georissa*, at least if one wishes for the taxonomy to reflect morphology as well. Since most species are allopatric, and therefore the maintenance of species barriers can usually not be tested, we present our taxonomy as a compromise, which remains to be further tested by future workers.

## Taxonomy

### Systematics and descriptions

#### Class Gastropoda Cuvier,1797

##### Family Hydrocenidae Troschel, 1856

###### Genus *Georissa* Blanford, 1864: 463

####### “Scaly group”

We here define an informal group of 13 species of *Georissa* from Malaysian Borneo that are characterised by one or more spiral rows of scale-like sculptures. As far as they were known at the time, our “scaly group” corresponds to Thompson and Dance’s (1983) “*hosei* group” + “*borneensis* group” p.p.

**Conchological description of a generalised “scaly group” representative**. *Protoconch*. Color (in living or freshly dead specimens): yellow, orange, red or brown. Sculpture: smooth, meshed, mixed or undefined. *Teleoconch*. Color (in living or freshly dead specimens): yellow, orange, red or brown. First whorl: convex, rounded to flat or angular. Subsequent whorls: convex, rounded, concave or tilted at the periphery, or flat, with well-impressed suture. Number of whorls: 2–3 ¼. Shell height (SH) (based on our conchological measurements of available studied materials stated in the methodology): 0.94–2.91 mm. Shell width (SW): 0.98–2.19 mm. Shell index (SI=SH/SW): 0.88-1.37. *Shell sculpture*. Radial sculpture: either absent or present. Growth lines: weak to strong. Spiral sculpture: absent or present; if present then weakly to strongly sculpted, continuous or discontinuous. Scales: between one and four spiral rows of vertical scales (any one of which may be more or less strongly pronounced than the others); scales can be minute to broad, low to acutely projecting. *Columella wall*. Smooth, translucent, and covering the umbilicus region. *Aperture*. Shape: oval to rounded, with straight to concave or convex parietal site, palatal edge either contiguous with or removed from the body whorl. Aperture height (AH): 0.50–1.33 mm. Aperture width (AW): 0.69–1.48 mm. Aperture index (AI=AH/AW): 0.65–1.02. *Peristome*. Simple, thickened inside, sharp toward the edge of the aperture. *Operculum*. Oval to rounded, with a peg facing inward, inner surface of the operculum has a small crater-like hole. Peg: straight or curved. The shell measurement of all measured “scaly group” *Georissa* are summarised in Suppl. material 4.

**Anatomy**. Haase and Schilthuizen (2007) described the anatomy of two closely related *Georissa*, viz. *G. saulae* and *G. filiasaulae*, and noted interspecific differences in radula, genital anatomy. Anatomical details of other “scaly group” representatives will be the focus of future studies and are not included in the present review.

**Habitat and ecology**. Members of the “scaly group” of *Georissa* live on limestone rocks, especially in wet and shaded environments. They are also found at lower density on dry limestone rocks, and occasionally on the limestone walls in cave systems (Haase and Schilthuizen 2007).

**Distribution**. There are at least nine species of this group distributed in Sarawak, and another four are distributed in Sabah (see Figs 3 and 4). In the distribution maps, we combined the geographical coordinates of each species from the known previous fieldwork locations and the available data from the collection repositories. The distribution of “scaly group” *Georissa* was assigned based on the available locality data from the collection from NHMUK, RMNH, ZMA, BOR, ZMU, and JJV. Localities may contain Malay words, namely: Batu = rock; Bukit = hill; Gua = cave; Gunung = mountain. We provide two distribution maps (Figs 3 and 4) to avoid overlapping of species that co-occur at the same or nearby locations.

In the following systematic descriptions of “scaly” *Georissa*, the species are arranged based on the molecular phylogeny. *Georissa scalinella* (van Benthem-Jutting, 1966), for which no genetic data are available, is placed at the top of the list.

For the stacked images of the “scaly” *Georissa* (Figs 5–17A–C), we decided not to remove the periostracum layers of the shells to retain the morphological characters of each species.

Since we needed fresh material to connect the morphology and molecular phylogenetics, we confined our study to Malaysian part of Borneo. We are aware that there might be species or populations in other parts of Borneo (Kalimantan, Indonesian Borneo and Brunei) which belong inside the “scaly group”. However, we hope that our study will stimulate colleagues that study *Georissa* in Kalimantan or Brunei to compare their material with our analysis.

**Figure 2.**
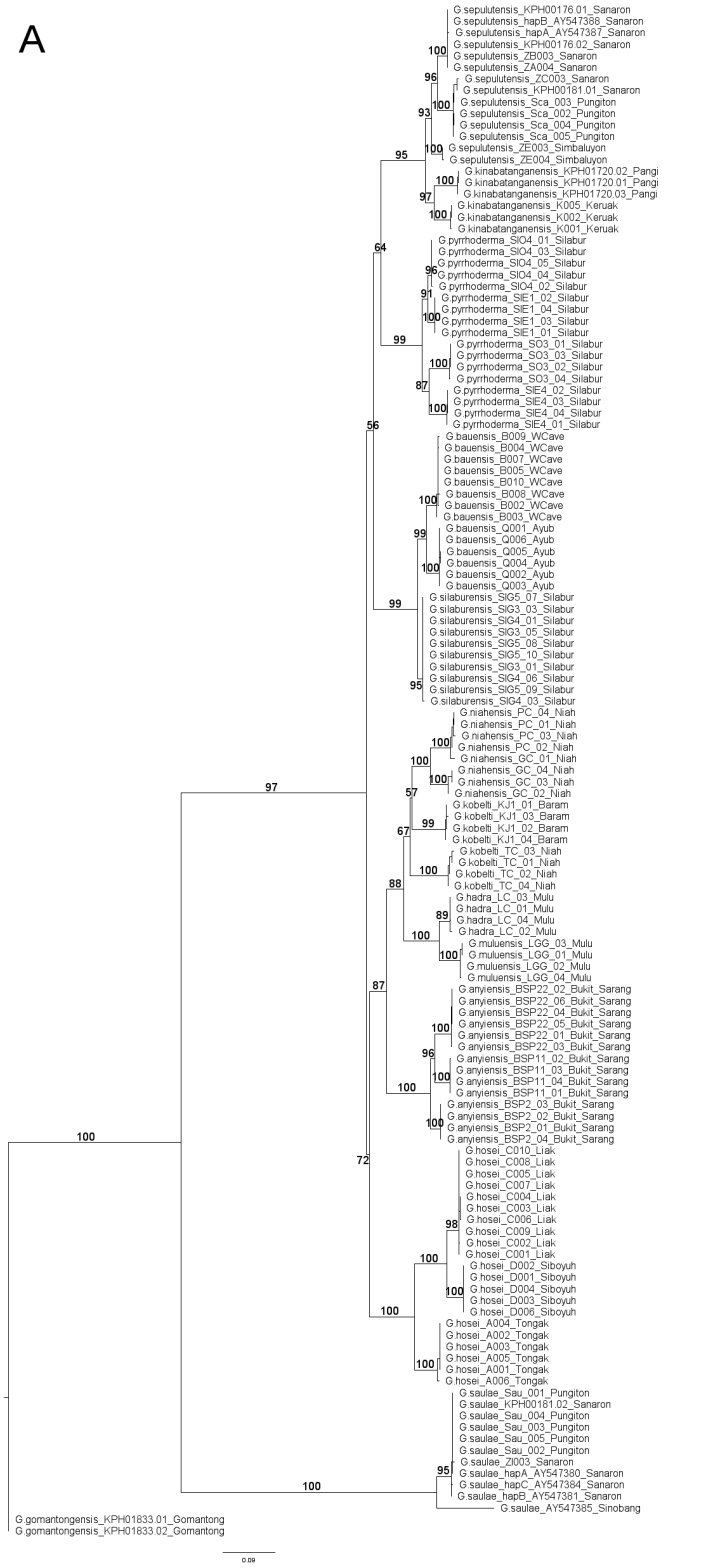
**A** Phylogeny from ML analysis with ultrafast bootstrapping (1000 replicates) **B** Phylogeny from MrBayes analysis. Analyses were conducted using concatenated sequence alignments of partial CO1 and 16S mtDNA of 133 individuals of “scaly” *Georissa* from Malaysian Borneo, with *Georissa gomantongensis* Smith, 1893 as the outgroup.

**Figure 3.**
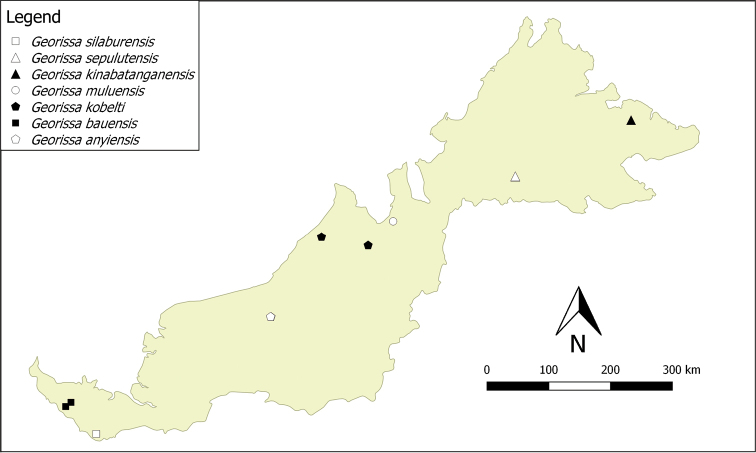
Distribution of seven “scaly group” *Georissa* species in Malaysian Borneo (based on the materials examined from NHM, RMNH, ZMA, BORN, MZU, and JJV).

**Figure 4.**
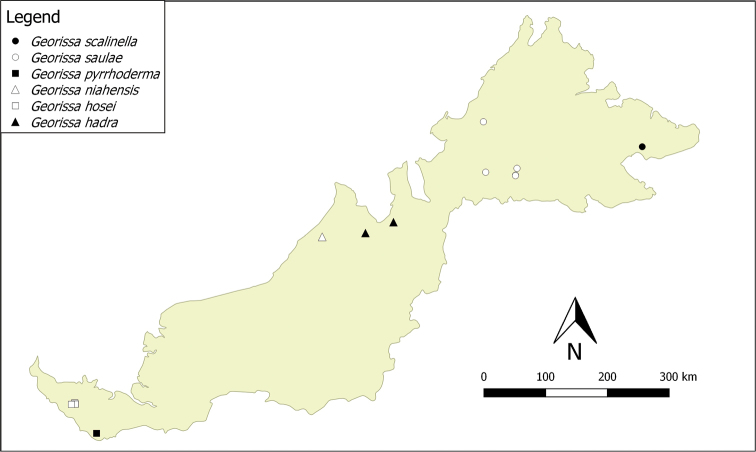
Distribution of five “scaly group” *Georissa* species in Malaysian Borneo (based on the materials examined from NHM, RMNH, ZMA, BORN, MZU, and JJV).

######## Georissa scalinella

Taxon classificationAnimaliaORDOFAMILIA

(van Benthem-Jutting, 1966)

Hydrocena scalinella van Benthem-Jutting, 1966: 39, fig. 1; Saul 1967: 108.
Georissa scalinella (van Benthem-Jutting): Thompson and Dance 1983: 119; Phung et al. 2017: 68, fig. 8B.

######### Type locality.

Lahad Datu Caves on Teck Guan Estate, Sabah.

######### Type material.

*Holotype*. Lahad Datu Caves on Teck Guan Estate, Sabah: ZMA/MOLL 135736 (seen). *Paratypes*. Lahad Datu Caves on Teck Guan Estate, Sabah: ZMA/MOLL 135735 (seen), ZMA/MOLL 315596 (seen).

######### Description.

*Protoconch*. Color: orange to red. Sculpture: smooth to meshed – semi oval mesh to undefined mesh pattern. Mesh width: 7–17 µm. *Teleoconch*. Color: orange. First whorl: flat at the shoulder. Subsequent whorls: flat above, slightly rounded below the periphery. Total number of whorls: 2 ¼-2 ½. SH: 1.56–1.80 mm, SW: 1.46–1.65 mm, SI: 1.03–1.15. *Shell sculpture*. Radial sculpture: absent, only weak to strong growth lines are visible. Spiral sculpture: present, and strongly sculpted, with continuous and discontinuous ribbing. Scales: a series of acute scales, low to highly projected, and regularly spaced. Intercept between growth lines and spiral ribbings form small pointed scale structures throughout the length of the body whorl. *Aperture*. Shape: oval. Basal side: rounded, angular at the columellar region. Parietal side: straight, palatal edge attached to the body whorl. AH: 0.78–0.94 mm, AW: 0.97–1.12 mm, AI: 0.75–0.89. *Holotype dimension*. SH: 1.88 mm, SW: 1.72 mm, AH: 0.84 mm, AW: 1.18 mm.

**Figure 5.**
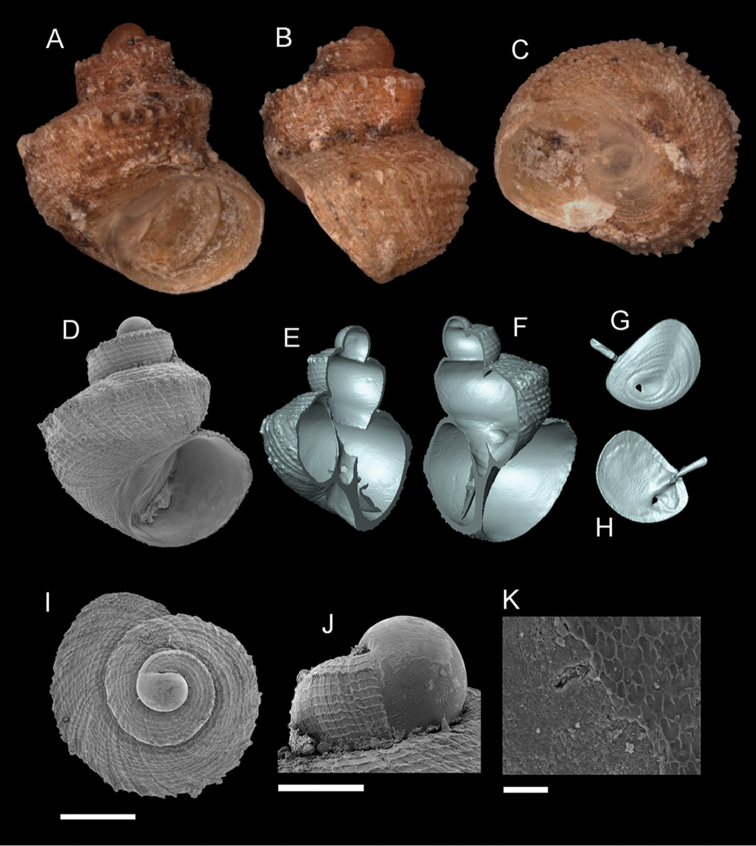
*Georissa scalinella* (van Benthem-Jutting, 1966). **A–C** Holotype: ZMA/MOL/ 135736 **D–K** Paratypes: ZMA/MOLL 135735. **A, D** Shell apertural view **B** Shell side view **C** Shell rear view **E–F** Shell cross-section from 3D model **G–H** Operculum frontal and ventral view **I** Shell top view **J** Protoconch side view **K** Close up of protoconch from top at 1000× magnification. Scale bars: 500 µm (**A–I**); 200 µm (**J**); 10 µm (**K**).

######### Cross diagnosis.

*Georissa scalinella* has a series of scales at the shoulder. In habitus and scale characters, it resembles *G. pyrrhoderma* from Gunung Silabur, Sarawak. The angular shoulder and small scale-like nodular structure at the intersection of strong spiral ribbings and growth lines are diagnostic for *G. scalinella*.

######### Distribution.

Known only from the type locality, Teck Guan Estate, Lahad Datu, Sabah, and also reported by Phung et al. (2017) at Pulau Tiga, Sandakan, Sabah. However, this may also refer to one of the other “scaly group” species from Sabah.

######### Discussion.

*Georissa scalinella* was first described as *Hydrocena scalinella* van Benthem-Jutting, 1966, before reclassified as *Georissa* by Thompson and Dance (1983). van Benthem-Jutting (1966) described *G. scalinella* as having strong spiral ribbing and multiple lines of scales.

######## Georissa saulae

Taxon classificationAnimaliaORDOFAMILIA

(van Benthem-Jutting, 1966)

Hydrocena saulae van Benthem-Jutting, 1966: 40, fig. 2; Saul 1967: 109.
Georissa saulae (van Benthem-Jutting): Thompson and Dance 1983: 118, fig. 29, 53–54; Haase and Schilthuizen 2007: 217, fig. 2; Clements 2008: 2762; Schilthuizen et al. 2012: 278; Beron 2015: 181; Phung et al. 2017: 68, fig. 8; Osikowski et al. 2017: 80.

######### Type locality.

Malaysia, Borneo, Sabah, Laying cave, Keningau.

######### Type material.

*Holotype*. Malaysia, Borneo, Sabah, Laying cave, Keningau: ZMA/MOLL 135731 (seen). *Paratypes*. Malaysia, Borneo, Sabah, Laying cave, Keningau: ZMA/MOLL 135598 (seen), ZMA/MOLL 135599 (seen).

######### Other material.

Simbaluyon limestone hill, Sabah, Malaysia: RMNH/MOL 333913, RMNH/MOL 333919. Crocker Range National Park, Gua Laing, Keningau, Sabah (05°29.00'N, 116°08.00'E): RMNH/MOL 335180, ZMA/MOLL 315592, ZMA/MOLL 315593, JJV 1119. Sepulut Valley, Gua Pungiton, Sabah (04°42.41'N, 116°36.04'E): BOR/MOL 28, BOR/MOL 12770, JJV 7544. Sepulut valley, Gua Sanaron, Sabah (04°42.05'N, 116°36.01'E): BOR/MOL 29, BOR/MOL 32, BOR/MOL 3493, JJV 7660. Pinangah valley, Batu Urun (Bukit Sinobang), Sabah (04°48.40'N, 116°37.35'E): BOR/MOL 31, JJV 1144, JJV 5632, JJV 7993. Mahua, Sabah: BOR/MOL 33. Pun Batu, Sepulut, Sabah (04°45.00'N, 116°10.00'E): JJV 1268. Sepulut valley, Batu Punggul, Sabah: JJV 1904.

######### Description.

*Protoconch*. Color: red to brown. Sculpture: meshed – ellipsoidal mesh pattern. Mesh width: 29–54 µm. *Teleoconch*. Color: brown to red. First whorl: convex to rounded. Subsequent whorls: convex to rounded. SH: 1.32–1.86 mm, SW: 1.14–1.48 mm, SI: 1.12–1.26. Total number of whorls: 2 ½-3 ¼. *Shell sculpture*. Radial sculpture: often present, when formed by vertical connections between corresponding scales on successive spiral ribs. These vertical connections, especially on the first whorls, form evenly spaced ribs that are raised when crossing a spiral rib. Spiral sculpture: present at the early teleoconch, subsequently becoming weaker, and later only short discontinuous lines are visible in between the radial sculptures. Scales: usually three or four discontinuous series of vertical, low to high-projecting scales, broad to pointed (only if the spiral series of scales are discontinuous). *Aperture*. Shape: rounded to slightly oval. Basal side: rounded, slightly angular before the columellar region. Parietal side: straight, connected to the palatal edge. AH: 0.58–0.83 mm, AW: 0.70–0.94 mm, AI: 0.76–0.92. *Holotype dimension*. SH: 1.60 mm, SW: 1.28 mm, AH: 0.66 mm, AW: 0.80 mm.

**Figure 6.**
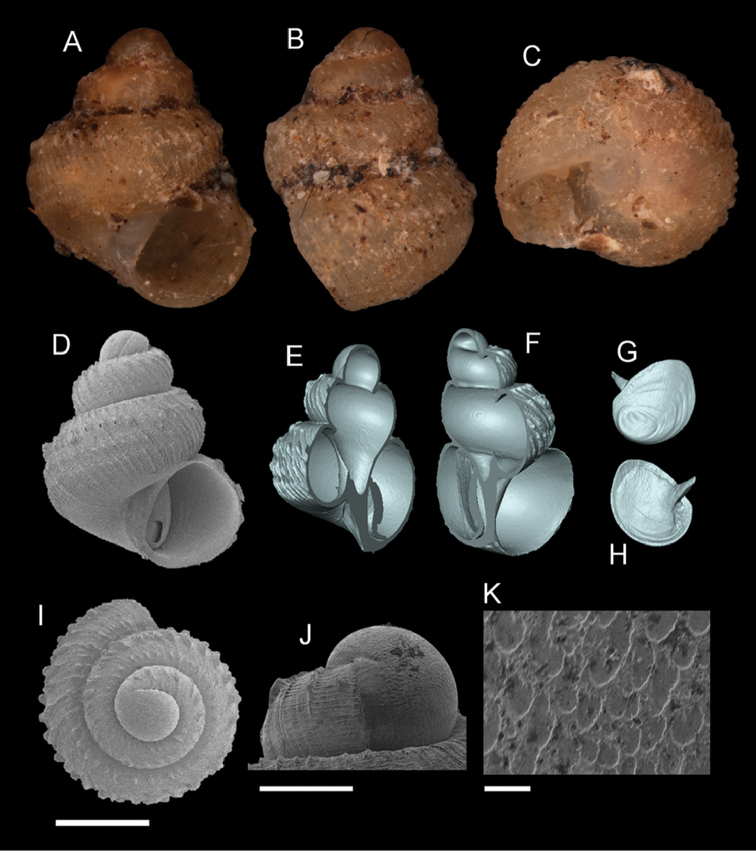
*Georissa saulae* (van Benthem-Jutting, 1966). **A–C** Holotype: ZMA/MOL 135599 **D–K** BOR/MOL 3493. **A, D** Shell apertural view **B** Shell side view **C** Shell rear view **E–F** Shell cross-section from 3D model **G–H** Operculum frontal and ventral view **I** Shell top view **J** Protoconch side view **K** Close up of protoconch from top at 1000× magnification. Scale bars: 500 µm (**A–I**); 200 µm (**J**); 10 µm (**K**).

######### Cross diagnosis.

*Georissa saulae* possesses clear diagnostic shell characters for distinction from other “scaly” *Georissa* species. *G. saulae* lacks a clear formation of spiral ribbing: although the spiral arrangement of the scales gives the impression of spiral sculpture, no underlying ribs are discernable. *G. scalinella*, *G. kinabatanganensis*, and *G. hosei*, on the other hand, have clear diagnostic spiral ribs. The shell whorls of *G. saulae* are broad but not as rapidly expanding as in *G. hosei*, *G. scalinella* or *G. kinabatanganensis*. It can also be distinguished from *G. scalinella* and *G. hosei* on the basis of a more elongate-conical shell shape and the aperture shape that is more rounded rather than oval.

######### Distribution.

The type locality of *Georissa saulae* is Laying cave, in the Crocker Range, Keningau, Sabah (a misspelling of Laing cave). Otherwise known from limestone outcrops in Sabah’s interior, viz., Simbaluyon, Sinobang, Sanaron, and Pungiton, and also has been recorded from Mahua, Sabah, which is not a limestone area. Phung et al. (2017) also report it from Pulau Tiga, Sabah.

######### Molecular analysis.

ML and Bayesian analyses show *Georissa saulae* (16S: n = 11) as a monophyletic group with 100% BS and 100% PP. Schilthuizen et al. (2012) reported that *G. saulae* is a paraphyletic group from which emerges the cave-dwelling species *G. filiasaulae* (Haase and Schilthuizen 2007), a fully unsculptured species that was not included in the present study. *G. saulae* + *G. filiasaulae* are sister to all other species in the “scaly group” (unpublished data).

######### Discussion.

*Georissa saulae* was initially described as *Hydrocena saulae* van Benthem-Jutting, 1966, then assigned to the genus *Georissa* by Thompson and Dance (1983). Thompson and Dance (1983) compared *G. saulae* with *G. scalinella*, and even suggested *G. saulae* might be a subspecies. In contrast, we find that *G. saulae* is a proper species with very distinct conchological characters, especially the presence of radial ribs on the shell, which makes it easy to identify. In some specimens from the entrance of the Batu Sanaron cave system, the vertical scales are spaced, and radial sculpture is weak. Such individuals presumably represent the hybrid zone with the cave-dwelling *G. filiasaulae* (Haase and Schilthuizen 2007, Schilthuizen et al. 2012).

######## Georissa hosei

Taxon classificationAnimaliaORDOFAMILIA

Godwin-Austen, 1889

Georissa hosei Godwin-Austen, 1889: 353, fig. 11 plate XXXIX; Smith 1893: 351, fig. 27 plate XXV; Thompson and Dance 1983: 116.

######### Type locality.

Borneo. (Unspecified)

######### Type material.

*Lectotype* (Designated by Thompson and Dance 1983). Borneo: NHMUK 1889.12.7.72 (glued on paper) (seen).

######### Other material.

Jambusan, North Borneo: NHMUK 92.7.20.122, NHMUK 92.7.23.33-4. Gunung Liak/Padang, Kampung Skiat Baru, Jambusan, Sarawak (01°24.05'N, 110°11.19'E): MZU/MOL 16.04, MZU/MOL 16.05, MZU/MOL 16.06, MZU/MOL 16.07. Bukit Siboyuh, Kampung Skiat Baru, Jambusan, Sarawak (01°22.90'N, 110°11.69'E): MZU/MOL 16.08. Bukit Tongak, Bidi, Bau/Jambusan, Sarawak (01°22.67'N, 110°08.32'E): MZU/MOL 16.09.

######### Description.

*Protoconch*. Color: red. Sculpture pattern: smooth. *Teleoconch*. Color: orange to red. First whorl: rounded or shouldered with flat surfaces above and below the shoulder. Subsequent whorls: convex to rounded; number of whorls: 2–2 ¼. SH: 1.06–1.55 mm, SW: 1.09–1.60 mm, SI: 0.94–1.12. *Shell sculpture*. Radial sculpture: absent, only weak growth lines. Spiral sculpture: present, weakly sculpted, continuous ribs, more prominent at the periphery. Scales: two to four series of low and broad vertical scales, regularly spaced, the upper scale series always the strongest, weaker series appear later at the spire, and the spaces between series are irregular. *Aperture*. Shape: oval. Basal side: rounded, angular at the columellar region. Parietal side: straight, palatal edge attached or removed at the body whorl. AH: 0.60–0.95 mm, AW: 0.80–1.16 mm, AI: 0.74–0.88.

**Figure 7.**
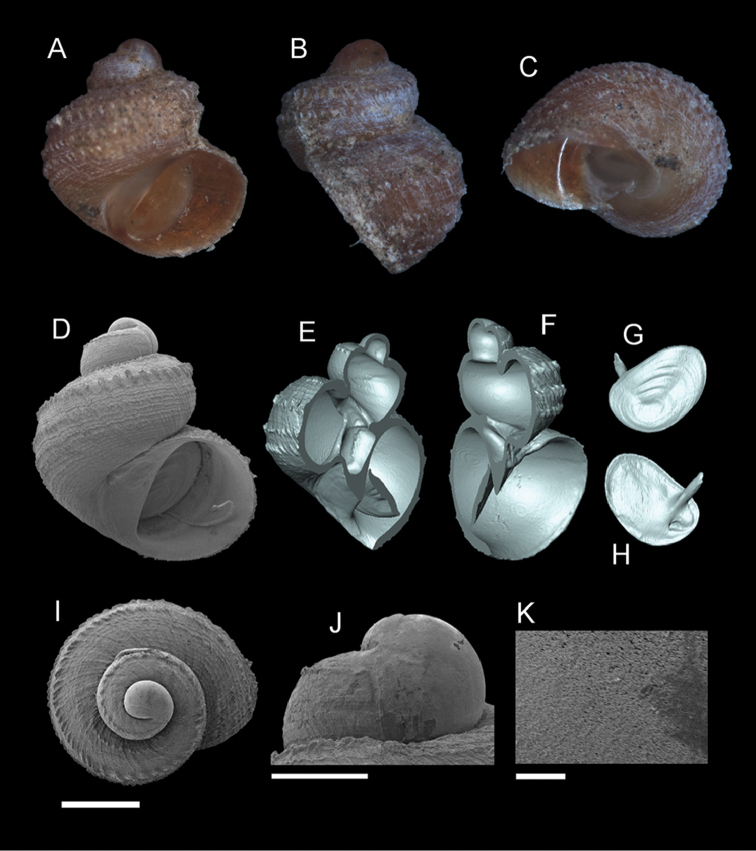
*Georissa hosei* Godwin-Austen, 1889. **A–C** MZU/MOL 16.05 **D–K** MZU/MOL 16.04. **A, D** Shell apertural view **B** Shell side view **C** Shell rear view **E–F** Shell cross-section from 3D model **G–H** Operculum frontal and ventral view **I** Shell top view **J** Protoconch side view **K** Close up of protoconch from top at 1000× magnification. Scale bars: 500 µm (**A–I**); 200 µm (**J**); 10 µm (**K**).

######### Cross diagnosis.

*Georissa hosei* has a diagnostic smooth protoconch. It possesses similar shell habitus and scale characters as *G. sepulutensis*, *G. pyrrhoderma*, and *G. kobelti*. However, the scales of *G. hosei* are rarely developed into large and acutely projected scales.

######### Distribution.

Known from Gunung Liak/Padang and Bukit Siboyuh at Kampung Skiat Baru, Jambusan, and Bukit Tongak, in the area of Bau, which is close to Jambusan.

######### Molecular analysis.

ML and Bayesian analyses shows that all *G. hosei* individuals (16S: n = 21; CO1: n = 11) group together in one clade with 100% BS and 100% PP, which is the sister group of all other “scaly group” species, except *G. saulae*.

######### Discussion.

Godwin-Austen (1889), when he described the species, mentioned that the sides of the spire (whorls) are flat, which we find to be the case for the first whorl in our material (as well as in the lectotype). The exact type locality was not specified, but Smith (1893) reported that the specimens of *G. hosei* described by Godwin-Austen (1889) were from Jambusan, Sarawak. It has to be noted that *G. hosei* is highly variable in shell shape and sculpture, even within a local population. For example, material we collected at Gunung Liak/Padang have anything between two and four series of broad and low scales. Material from Bukit Tongak has three to four spiral threads with scales. Material from Bukit Siboyuh, finally, is brighter in color (orange), with only one or two spiral series of scales. These three limestone outcrops are all within the area of not more than 10 km radius. Thompson and Dance (1983) noted that *G. hosei* is widely distributed in Sarawak, and they give Baram, Marudi, Niah, Tatau, and Bukit Sarang as localities. However, as we elaborate in this paper, many of these populations are not conspecific with *G. hosei*. For example, the image of “*G. hosei*” provided by Thompson and Dance (1983) – UF 35919, from Batu Gading, Baram, appears conspecific to *G. kobelti*. Also, their “*G. hosei*” from Bukit Sarang we here describe as a *G. anyiensis* sp. n.

######## Georissa anyiensis sp. n.

Taxon classificationAnimaliaORDOFAMILIA

http://zoobank.org/DD0DD84B-0363-4B68-9A93-877E3602DAE3

Georissa hosei Godwin-Austen: Thompson and Dance 1983: 117, materials from Tatau Valley, Bukit Sarang, Bintulu, Sarawak. (**non**G. hosei Goodwin-Austen, 1889)

######### Type locality.

Bukit Anyi at Bukit Sarang, Bintulu, Sarawak, Malaysia (02°39.25'N, 113°02.72'E).

######### Type material.

*Holotype*. Bukit Anyi at Bukit Sarang, Bintulu, Sarawak, Malaysia (02°39.25'N, 113°02.72'E): MZU/MOL 17.90 (leg. MZ Khalik and SK Reduan). *Paratypes*. Bukit Anyi at Bukit Sarang, Bintulu, Sarawak (02°39.25'N, 113°02.72'E): MZU/MOL 17.53, MZU/MOL 17.54, MZU/MOL 17.55, MZU/MOL 17.56, MZU/MOL 17.57, MZU/MOL 17.58, MZU/MOL 17.59, MZU/MOL 17.60, MZU/MOL 17.61, JJV 12840 (40), JJV 12841 (1). Bukit Lebik at Bukit Sarang, Bintulu, Sarawak (02°39.32'N, 113°02.43'E): MZU/MOL 17.50, MZU/MOL 17.51, MZU/MOL 17.52, JJV 12842 (20), JJV 12843 (1). From Thompson and Dance 1983, Bukit Sarang, Tatau valley (20°45'N, 113°02'E): UF 35914, UF 35915, UF 35921 (not seen). Each lot of examined paratypes from MZU are more than 50 individuals.

######### Etymology.

Named after the hill Bukit Anyi at Bukit Sarang, Bintulu, Sarawak, Malaysia, the type locality.

######### Description.

*Protoconch*. Color: yellow to orange. Sculpture pattern: meshed – rounded to ellipsoidal or undefined mesh shape. Mesh width: 8–30 µm. *Teleoconch*. Color: yellow. First whorl: shouldered, cylindrical. Subsequent whorls: convex to rounded, with a deeply impressed suture. SH: 1.39–1.98 mm, SW: 1.32–1.72 mm, SI: 1.05–1.08. Total number of whorls: 2 ¼–2 ¾. *Shell sculpture*. Radial sculpture: absent, only weak to strong growth lines are visible. Spiral sculpture: present, strongly sculpted, continuous ribs, more prominent at the periphery. Scales: at the shoulder a continuous spiral row of highly projecting diagonal crown-like scales; subordinate to that, three to four series of tall, broad or acute diagonal scales, regularly spaced, the uppermost of these always stronger than the lower ones, inter-series pacing irregular. *Aperture*. Shape: oval to rounded. Basal side: rounded, angular at the columellar region. Parietal side: straight. AH: 0.67–0.91 mm, AW: 0.90–1.17 mm, AI: 0.74–0.93. *Holotype dimension*. SH: 1.91 mm, SW: 1.72 mm, AH: 0.90 mm, AW: 1.14 mm.

**Figure 8.**
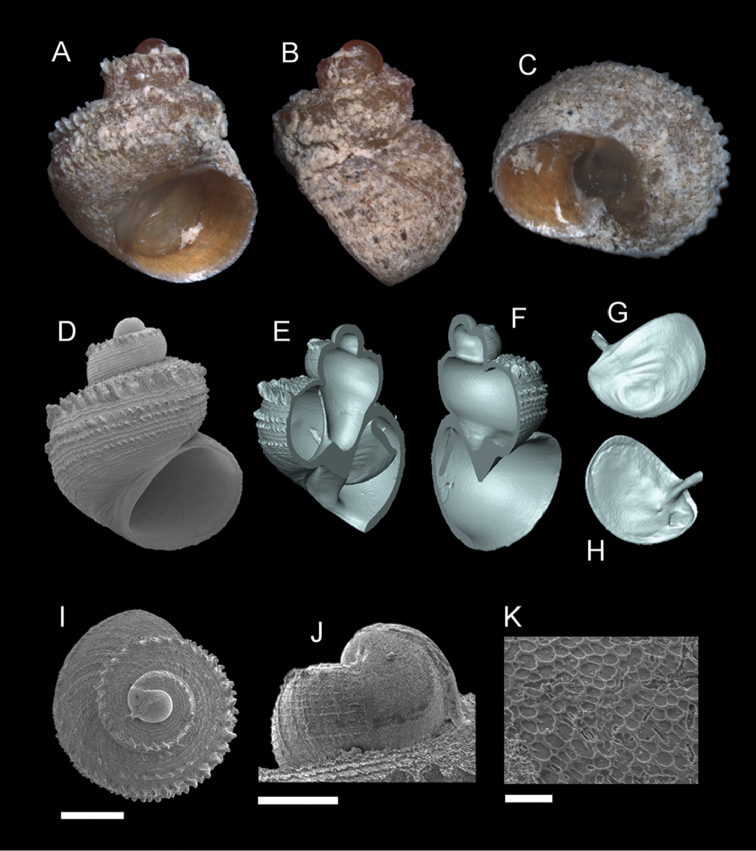
*Georissa anyiensis* sp. n. **A–C** Holotype: MZU/MOL 17.90 **D–K** Paratypes: MZU/MOL 17.55. **A, D** Shell apertural view **B** Shell side view **C** Shell rear view **E–F** Shell cross-section from 3D model **G–H** Operculum frontal and ventral view **I** Shell top view **J** Protoconch side view **K** Close up of protoconch from top at 1000× magnification. Scale bars: 500 µm (**A–I**); 200 µm (**J**); 10 µm (**K**).

######### Cross diagnosis.

In general, *G. anyiensis* has a shell shape that is similar to *G. kobelti*, *G. scalinella*, and *G. muluensis*. However, *G. anyiensis* has an extremely prominent, crown-like spiral series of large scales on the shell periphery, which distinguishes it from other “scaly” *Georissa*.

######### Distribution.

Known from Bukit Anyi and Bukit Lebik, two isolated hills at Bukit Sarang, Bintulu, Sarawak.

######### Molecular analysis.

ML and Bayesian analyses show that the *G. anyiensis* individuals (16S: n = 13; CO1: n = 12) form a monophyletic group with 100% BS and 100% PP, sister group to the four species *G. niahensis* + *G. kobelti* + *G. hadra* + *G. muluensis*.

######## Georissa muluensis sp. n.

Taxon classificationAnimaliaORDOFAMILIA

http://zoobank.org/8CAE7706-39F4-47ED-9275-7606DDD5FC26

######### Type locality.

Lagang Cave, Mulu National Park, Mulu, Sarawak, Malaysia (04°03.06'N, 114°49.37'E).

######### Type material.

*Holotype*. Lagang Cave, Mulu National Park, Mulu, Sarawak, Malaysia (04°03.06'N, 114°49.37'E): MZU/MOL 17.86 (leg. MZ Khalik and SK Reduan). *Paratypes*. Lagang Cave, Mulu National Park, Mulu, Sarawak (04°03.06'N, 114°49.37'E): MZU/MOL 17.30 (13), MZU/MOL 17.31 (9).

######### Other material.

Deer Cave, Mulu National Park, Mulu, Sarawak: JJV 10533 (this sample, approximately 120 individuals, also contains specimens of *G. hadra*), JJV 10554 (this sample contains 5 individual of *G. muluensis*, 1 individual *G. hadra*), JJV 10533 (this sample, approximately 150 individuals, also contains specimens of *G. hadra* and *G. kobelti*). Mulu N.P., Mulu, Sarawak: JJV 10527.

######### Etymology.

Named after Mulu National Park, Sarawak, Malaysia, the type locality.

######### Description.

*Protoconch*. Color: yellow. Sculpture pattern: meshed – ellipsoidal mesh shape. Mesh width: 16–26 µm. *Teleoconch*. Color: yellow. First whorl: shouldered, above the shoulder flat, nearly horizontal; below the shoulder flat, cylindrical, but abruptly withdrawn into the deeply incised suture. Subsequent whorls: convex to rounded. SH: 1.67–2.05 mm, SW: 1.57–1.79 mm, SI: 1.08–1.18. Total number of whorls: 2 ¼-3. *Shell sculpture*. Radial sculpture: absent, only weak to strong growth lines are visible. Spiral sculpture: present, consisting of thin, but strongly sculpted and continuous ribs. Scales: two to three series of tall and diagonal scales, regularly spaced, the upper scale series always stronger than the lower ones, weaker series appear later at the spire or consist only of randomly spaced arrays of acute nodules, widely spaced between the first and second scale series, more densely spaced later. *Aperture*. Shape: rounded to slightly oval. Basal side: rounded, angular at the columellar region. Parietal side: straight to slightly curved. AH: 0.82–0.98 mm, AW: 1.03–1.18 mm, AI: 0.77–0.83. *Holotype dimension*. SH: 1.67 mm, SW: 1.53 mm, AH: 0.82 mm, AW: 1.07 mm.

**Figure 9.**
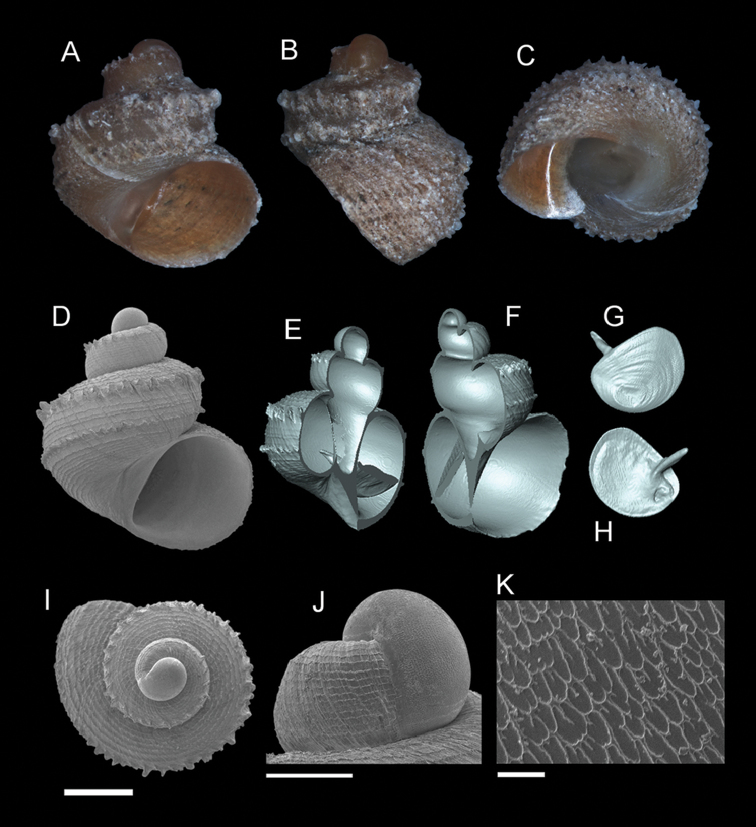
*Georissa muluensis* sp. n. **A–C** Holotype: MZU/MOL 17.86 **D–K** Paratypes: MZU/MOL 17.30. **A, D** Shell apertural view **B** Shell side view **C** Shell rear view **E–F** Shell cross-section from 3D model **G–H** Operculum frontal and ventral view **I** Shell top view **J** Protoconch side view. **K**. Close up of protoconch from top at 1000× magnification. Scale bars: 500 µm (**A–I**); 200 µm (**J**); 10 µm (**K**).

######### Cross diagnosis.

The wide spacing of the major spiral scale series of *G. muluensis* is similar to *G. kinabatanganensis*, but *G. muluensis* has a more elongated shell shape, rather than the more flattened habitus of *G. kinabatanganensis*. In general shell shape and sculpture *G. muluensis* also resembles *G. hadra*, which, however, is larger and more elongated.

######### Distribution.

Known only from the small area of Lagang Cave, Mulu National Park, Mulu, Sarawak, Malaysia.

######### Molecular analysis.

ML and Bayesian analyses show that the individuals of *G. muluensis* (16S: n = 4; CO1: n = 4) form a monophyletic group with 100% BS and 100% PP, which is the sister group of *G. hadra*.

######## Georissa hadra

Taxon classificationAnimaliaORDOFAMILIA

Thompson & Dance, 1983

Georissa hadra Thompson & Dance, 1983: 115–116, fig. 32, 43–46.

######### Type locality.

Butik Besungai, a small limestone hill 0.5 miles southwest of Batu Gading, and about 4 miles northeast of Long Lama, Baram Valley, Fourth Division, Sarawak. 03°52'N, 114°25'E.

######### Type material.

*Holotype*. Butik Besungai, a small limestone hill 0.5 miles southwest of Batu Gading, and about 4 miles northeast of Long Lama, Baram Valley, Fourth Division, Sarawak: UF36107 (not seen). *Paratypes*. Butik Besungai ½ mile SW. of Batu Gading, 4 miles NE. of Long Lama, Baram Valley, 4^th^ Div., Sarawak, Malaysia: BMNH 1984004 (seen). Baram valley, Long Lama, Bt. Besungai 0.5 m SW of Batu Gading, Sarawak (03°52.00'N, 114°25.00'E): JJV 13421 (seen).

######### Other material.

Lang Cave, Mulu N.P., Mulu, Sarawak (04°01.49'N, 114°49.48'E): MZU/MOL 17.32, MZU/MOL 17.33, MZU/MOL 17.34, MZU/MOL 17.35. Deer Cave, Mulu N.P., Mulu, Sarawak: JJV 10533 (this sample, approximately 120 individuals, also contains *G. muluensis*), JJV 10554 (5 individual of *G. muluensis*, 1 individual *G. hadra*), JJV 10533 (this sample, approximately 150 individuals, also contains *G. muluensis* and *G. kobelti*).

Butik = a misspelling of Bukit, a local name for hill.

######### Description.

*Protoconch*. Color: orange. Sculpture pattern: meshed – rounded to ellipsoidal or undefined mesh shape. Mesh width: 12–24 µm. *Teleoconch*. Color: orange. First whorl: with a distinct shoulder (provided with a series of minuscule scales), above the shoulder flat and tapering towards the suture, below the shoulder flat and cylindrical. Subsequent whorls: distinctly scalariform, with three separate aspects separated by two or more main spiral series of scales: above the uppermost spiral series gently curved towards the suture; in between both spiral series flat and cylindrical; below the lowest spiral series abruptly narrowed towards the deeply impressed suture (on the final whorl these three aspects fuse, forming a uniformly rounded impression). SH: 2.61–2.91 mm, SW: 2.05–2.19 mm, SI: 1.21–1.37. Total number of whorls: 2 ¾-3 ¼. *Shell sculpture*. Radial sculpture: absent, but with strong and unevenly layered growth lines. Spiral sculpture: present, weakly sculpted, continuous to discontinuous. Scales: two to four irregularly spaced series of low to high, and minute to broad diagonal scales, densely spaced, the first scale series always the strongest, weaker series appearing later at the spire. *Aperture*. Shape: rounded, with a tilt below the palatal side. Basal side: rounded, angular at the columellar region. Parietal side: straight to curved. AH: 1.11–1.33 mm, AW: 1.32–1.48 mm, AI: 0.83–1.01.

**Figure 10.**
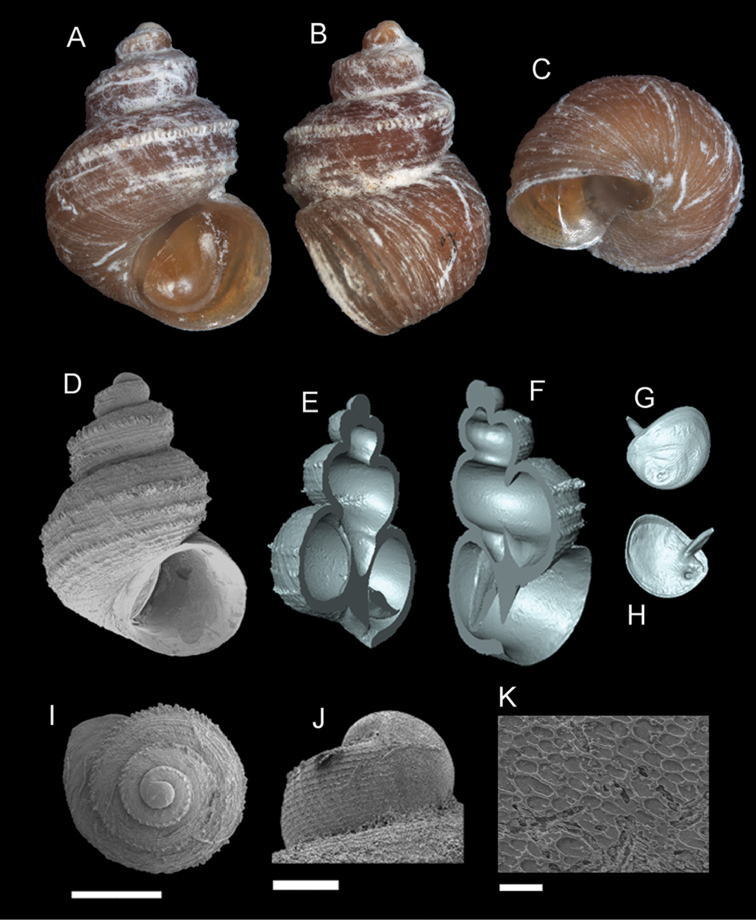
*Georissa hadra* Thompson & Dance, 1983. **A–C** MZU/MOL 17.33 **D–K** ZMA/MOLL 17.32. **A, D** Shell apertural view **B** Shell side view **C** Shell rear view **E–F** Shell cross-section from 3D model **G–H** Operculum frontal and ventral view **I** Shell top view **J** Protoconch side view **K** Close up of protoconch from top at 1000× magnification. Scale bars: 1 mm (**A–I**); 200 µm (**J**); 10 µm (**K**).

######### Cross diagnosis.

*Georissa hadra* has scales which are densely arranged, unlike *G. scalinella*, *G. hosei*, *G. muluensis*, *G. anyiensis*, and *G. kobelti*, which have more widely spaced scales. In shell shape, *G. hadra* is similar to the later three species but larger and more distinctly scalariform. *G. hadra* is similar in size to *G. niahensis*, but it has a more slender habitus and a more rounded periphery.

######### Distribution.

The type locality for *G. hadra* is Bukit Besungai, Baram, Sarawak. We also obtained it at Mulu, Sarawak. Currently, therefore, the known distribution range is restricted to Mulu and Baram.

######### Molecular analysis.

ML and Bayesian analyses retrieve *G. hadra* (16S: n = 4; CO1: n = 4) as a single clade with 89% BS and 100% PP, sister to *G. muluensis*.

######### Discussion.

The paratypes of Thompson and Dance (1983) have a very pale orange color, compared to recently collected materials from Mulu; presumably the color has faded.

######## Georissa kobelti

Taxon classificationAnimaliaORDOFAMILIA

Gredler, 1902

Georissa kobelti Gredler, 1902: 61; Zilch 1973: 265, fig. 11; Thompson and Dance 1983: 117, fig. 28, 50–52.
Georissa hosei Godwin-Austen: Thompson and Dance 1983: 117, fig. 47–49, material from Bukit Besungai at Baram Valley, Niah, Kejin trib. of Baram river (**non**G. hosei Goodwin-Austen, 1889).

######### Type locality.

Niah, Baram (Sarawak, Borneo). Unspecified.

######### Type material.

*Lectotype* (Designated by Zilch 1973). Niah, Baram (Sarawak, Borneo): SMF 215893a (not seen).

######### Other material.

Trade Cave, Niah National Park, Niah, Sarawak (03°49.13'N, 113°46.86'E): MZU/MOL 17.36. Great Cave, Niah National Park, Niah, Sarawak: MZU/MOL 17.37. Bukit Kaijin, Baram, Sarawak (03°41.75'N, 114°27.55'E): MZU/MOL 17.38, MZU/MOL 17.39, MZU/MOL 17.40, MZU/MOL 17.41, MZU/MOL 17.42, MZU/MOL 17.43, MZU/MOL 17.44, MZU/MOL 17.45, MZU/MOL 17.46, MZU/MOL 17.47, MZU/MOL 17.48, MZU/MOL 17.49, JJV 10217. Bukit Kasut, Niah N.P., Niah, Sarawak: JJV 10254. Niah N.P., Niah, Sarawak: JJV 1565, JJV 5466, JJV 10306, JJV 10392. Deer Cave, Mulu N.P., Mulu, Sarawak: JJV 10533 (the sample, approximately 150 individuals, also contains *G. muluensis* and *G. hadra*). Tatau river valley, Bukit Sarang, Bintulu, Sarawak: JJV 12551, JJV 12838. From Thompson and Dance 1983, Niah, Baram (Sarawak, Borneo): UF 35919, UF 36179 (not seen).

######### Description.

*Protoconch*. Color: orange to red. Sculpture: meshed – semi-oval mesh shape. Mesh width: 11–22 µm. *Teleoconch*. Color: ranging from red to yellow. First whorl: convex to rounded. Subsequent whorls: convex to rounded. SH: 1.75–2.11 mm, SW: 1.48–1.75 mm, SI: 1.18–1.28. Total number of whorls: 2 ¾-3. *Shell sculpture*. Radial sculpture: absent, only weak growth lines. Spiral sculpture: present with thin but strongly continuous spiral ribs, forming small acute scales near the suture. Scales: three to four spiral rows of tilted, nearly vertical scales, the upper series stronger than the lower ones, scale prominence ranging from high to low and from small and acute to broadly sculpted and ear-like. Scales are regularly spaced, as are the scale series themselves. *Aperture*. Shape: rounded to oval. Basal side: rounded, angular before the columellar region. Parietal side: curved. AH: 0.82–1.04 mm, AW: 1.02–1.17 mm, AI: 0.71–0.90.

**Figure 11.**
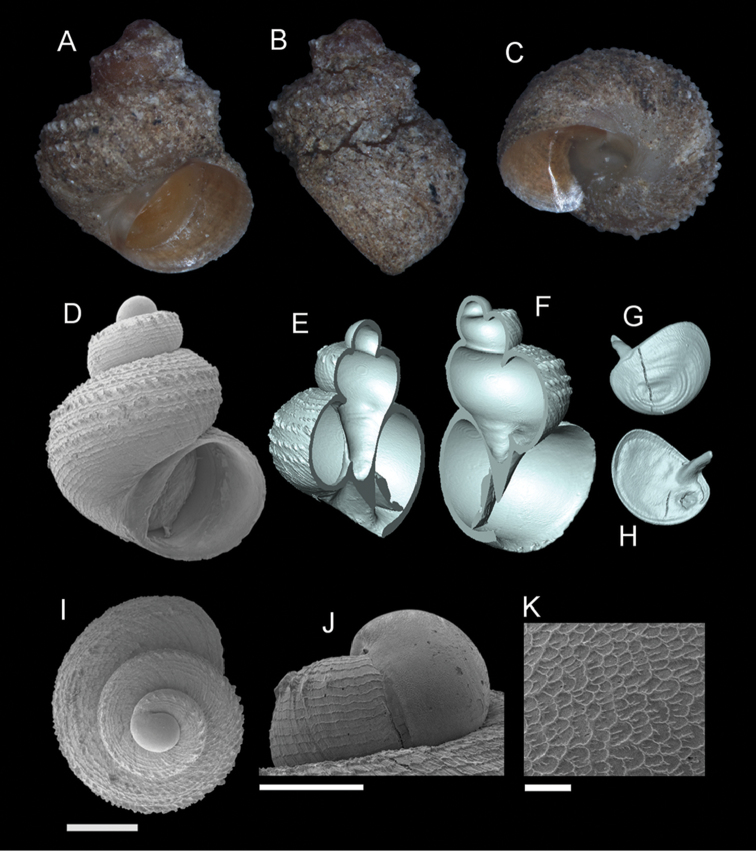
*Georissa kobelti* Gredler, 1902. **A–C** MZU/MOL 17.40 **D–K** MZU/MOL 17.38. **A, D** Shell apertural view **B** Shell side view **C** Shell rear view **E–F** Shell cross-section from 3D model **G–H** Operculum frontal and ventral view **I** Shell top view **J** Protoconch side view **K** Close up of protoconch from top at 1000× magnification. Scale bars: 500 µm (**A–I**); 200 µm (**J**); 10 µm (**K**).

######### Cross diagnosis.

The image of the *G. kobelti* lectotype by Zilch (1973) does not provide detailed information about the scale characters of *G. kobelti* as compared to the images of the individual from UF provided by Thompson and Dance (1983), which clearly shows the diagnostic characters of the ear-like scale pattern of this species. In shell habitus, *G. kobelti* is similar to some populations of *G. anyiensis*, *G. saulae*, and *G. hosei*, but these species differ from *G. kobelti* by the pattern of their diagonal scales.

######### Distribution.

The lectotype in Senckenberg (SMF 215893a) was obtained from an unspecified location. As far as known, the species is restricted to the area of Niah to Baram, northern Sarawak. Thompson and Dance (1983) also stated that they examined this species from Beluru, which is located between Niah and Baram.

######### Molecular analysis.

In the ML and Bayesian analyses of *G. kobelti* (16S: n = 8; CO1: n = 8), the Niah and Baram populations form highly supported clades (99% and 100% BS, respectively, and 100% PP for both clades), which are paraphyletic with respect to *G. niahensis*.

######## Georissa niahensis

Taxon classificationAnimaliaORDOFAMILIA

Godwin-Austen, 1889

Georissa niahensis Godwin-Austen, 1889: 353; Thompson and Dance 1983: 119.

######### Type locality.

Niah Hills, Borneo. (Unspecified)

######### Type material.

*Lectotype* (Designated by Thompson and Dance 1983). Niah Hills, Borneo: NHMUK 1889.12.7.69 (glued on paper) (seen). *Paralectotype*. Niah Hills, Borneo: NHMUK 1889.12.7.70 (glued on paper) (seen).

######### Other material.

Painted Cave, Niah National Park, Niah, Sarawak (03°48.68'N, 113°47.25'E): MZU/MOL 17.25.

######### Description.

*Protoconch*. Color: red. Sculpture pattern: smooth and meshed – ellipsoid to irregular mesh shape. Mesh width: 12–19 µm. *Teleoconch*. Color: orange to red. First whorl: curved above the shoulder, flat and cylindrical below the shoulder. Subsequent whorls: convex, angular at the periphery. SH: 1.81–2.53 mm, SW: 1.51–1.99 mm, SI: 1.10–1.29. Total number of whorls: 3–3 ¼. *Shell sculpture*. Radial sculpture: absent, only strong and unevenly layered growth lines. Spiral sculpture: present, strongly sculpted, continuous to discontinuous, well defined from the first whorl all the way to the peristome. Scales: a single spiral series of low and minute acute scales, regularly spaced at the first whorl, but weaker, grading to imperceptible on the body whorl. *Aperture*. Shape: rounded. Basal side: rounded, angular at the columellar region. Parietal side: straight to curved. AH: 0.85–1.24 mm, AW: 0.92–1.27 mm, AI: 0.83–1.02.

**Figure 12.**
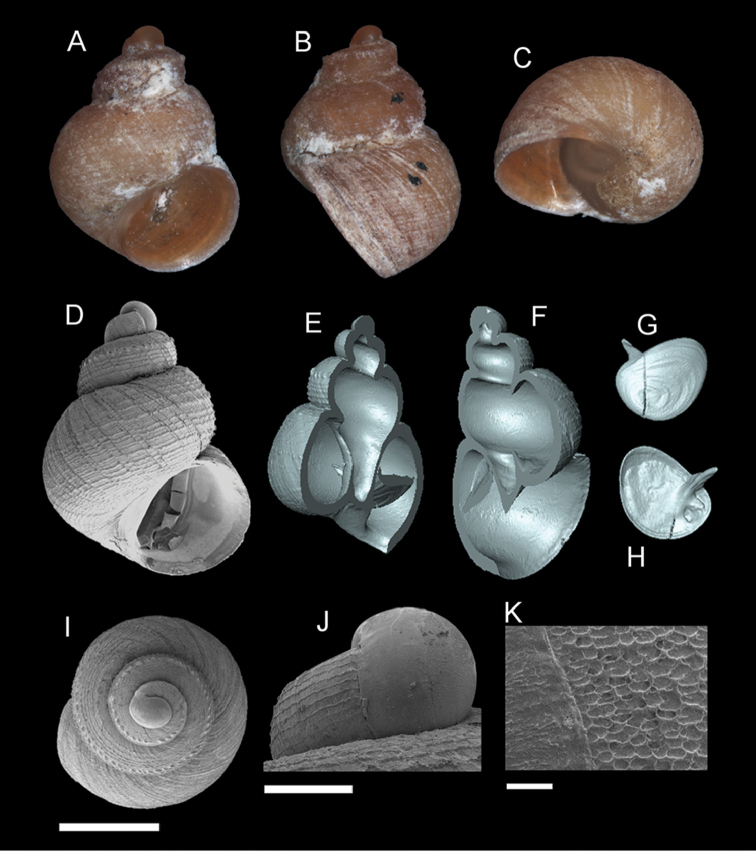
*Georissa niahensis* Godwin-Austen, 1889. **A–K** MZU/MOL 17.25 **A, D** Shell apertural view **B** Shell side view **C** Shell rear view **E–F** Shell cross-section from 3D model **G–H** Operculum frontal and ventral view **I** Shell top view **J** Protoconch side view **K** Close up of protoconch from top at 1000× magnification. Scale bars: 1 mm (**A–I**); 200 µm (**J**); 10 µm (**K**).

######### Cross diagnosis.

*Georissa niahensis* has a distinctive single series of small scales on the whorl shoulder, close to the suture. *G. niahensis* is one of the largest Bornean *Georissa*, in shell size only matched by *G. hadra* (which, however, is more slender, angular at the shoulder and has a flat to slightly rounded whorls). In general shell shape, *G. niahensis* is closest to *G. kobelti*, but the latter species is more rounded, while *G. niahensis* has a distinctly convex periphery.

######### Distribution.

Known to occur only at Niah, Sarawak.

######### Molecular analysis.

ML and Bayesian analyses of *G. niahensis* (16S: n = 8; CO1: n = 7) showed that all *G. niahensis* specimens form one clade with 100% BS and 100% PP. The sister group is the *G. kobelti* population from Baram (*G. kobelti* is paraphyletic).

######### Discussion.

Both Godwin-Austen (1889) and Thompson and Dance (1983) did not mention anything about the small scale-like nodules close to the suture of *G. niahensis*. Godwin-Austen (1889): “*Shell elongately conoid, solid, imperforate; sculpture a very in-distinct, ill-defined spiral liration, about 20 on the penultimate whorl, upon a rough surface crossed by transverse lines of growth; color ruddy ochre; spire high; apex pointed, finely papillated, minutely lirate; suture impressed; whorls 4 ½ convex; aperture oval, oblique; peristome simple, acute below; columellar margin straight*”. Thompson and Dance (1983): “*G. niahensis is similar in sculpture to G. williamsi but is much larger. G. niahensis also shows similarities to the hosei group in the depth of the suture and the relatively rapid expanding whorls, but it lacks the node-like sculpture found among species of that group.*” The scales are relatively small which are not very conspicuous among the strong growth lines, and this is the reason why in the previous description of the species the scale characters were lacking. Thompson and Dance (1983) compared *G. niahensis* with what they call the *hosei* group, based on the size and the deeply impressed suture.

######## Georissa silaburensis sp. n.

Taxon classificationAnimaliaORDOFAMILIA

http://zoobank.org/E88C99A8-8A0D-4438-9699-9AA86CAEE217

######### Type locality.

Gunong Silabur, Serian, Sarawak, Malaysia (00°57.28'N, 110°30.22'E).

######### Type material.

*Holotype*. Gunong Silabur, Serian, Sarawak, Malaysia (00°57.28'N, 110°30.22'E): MZU/MOL 17.88 (leg. MZ Khalik and SK Reduan). *Paratypes*. Gunong Silabur, Serian, Sarawak, Malaysia (00°57.28'N, 110°30.22'E): MZU/MOL 17.01, MZU/MOL 17.02, MZU/MOL 17.03, MZU/MOL 17.04, MZU/MOL 17.05, MZU/MOL 17.06, MZU/MOL 17.07, MZU/MOL 17.08. Borneo, Sarawak, First Division, western side of Gunong Selabor, Semabang entrance to Lobang Batu Cave (00°55'N, 110°25'E): NHMUK 1984005 (seen). Each lot of examined paratypes from MZU are more than 50 individuals.

######### Etymology.

Named after Gunung Silabur, Serian, Sarawak, Malaysia, the type locality.

######### Description.

*Protoconch*. Color: red. Sculpture pattern: meshed – round to irregular mesh pattern. Mesh width: 8–18 µm. *Teleoconch*. Color: red. First whorl: rounded. Subsequent whorls: convex, number of whorls: 2–2 ¼. SH: 1.59–1.99 mm, SW: 1.50–1.76 mm, SI: 1.06–1.13. *Shell sculpture*. Radial sculpture: absent or weak to strong growth lines. Spiral sculpture: present, thin but strongly sculpted, continuous ribs, more prominent at the periphery. Scales: two to six or more randomly sculpted series of low and broad horizontal scales, or else acute horizontal nodules on the spiral sculpture, scale series irregularly spaced, which series is the most prominent is not consistent across individuals. *Aperture*. Shape: rounded. Basal side: rounded, angular at the columellar region. Parietal side: straight, palatal edge attached to slightly removed from the body whorl. AH: 0.95–1.09 mm, AW: 1.00–1.17 mm, AI: 0.92–0.99. *Holotype dimension*. SH: 1.68 mm, SW: 1.53 mm, AH: 0.95 mm, AW: 1.09 mm.

**Figure 13.**
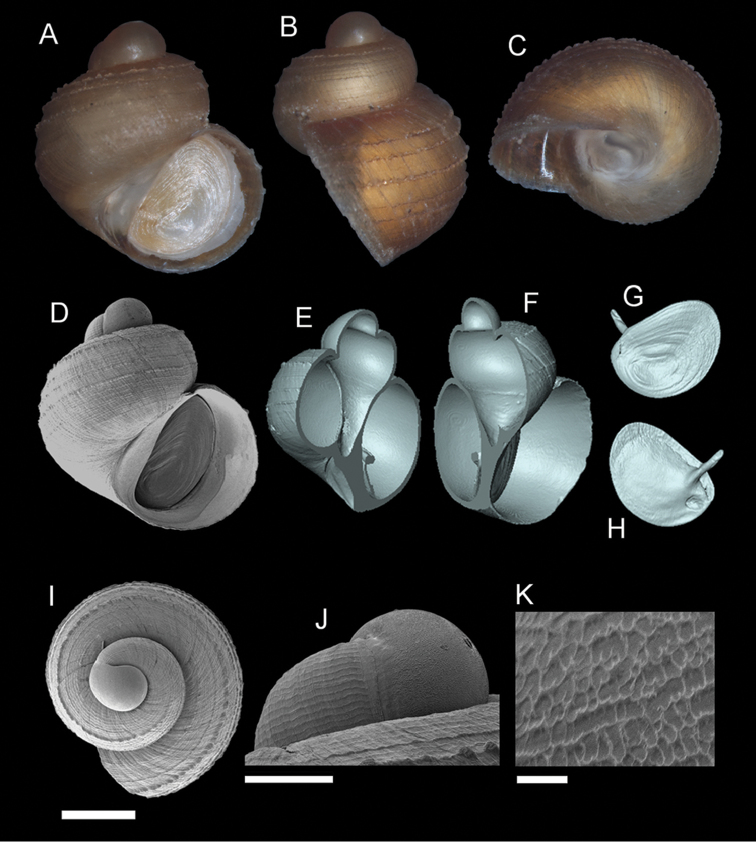
*Georissa silaburensis* sp. n. **A–C** Holotype: MZU/MOL 17.88 **D–K** Paratypes: MZU/MOL 17.04. **A, D** Shell apertural view **B** Shell side view **C** Shell rear view **E–F** Shell cross-section from 3D model **G–H** Operculum frontal and ventral view **I** Shell top view **J** Protoconch side view **K** Close up of protoconch from top at 1000× magnification. Scale bars: 500 µm (**A–I**); 200 µm (**J**); 10 µm (**K**).

######### Cross diagnosis.

The shell shape of *G. silaburensis* is distinct compared to other “scaly group” *Georissa*. It has rapid shell expansion like *G. hosei* and *G. scalinella*, but *G. silaburensis* has a different sculpture, consisting of horizontal, rather than vertical or diagonal scales. In addition, the whorls are rounded and convex, with the aperture almost circular, close to *G. saulae*.

######### Distribution.

Known from the inside of the cave system of Gunung Silabur, Serian, Sarawak.

######### Molecular analysis.

ML and Bayesian analyses show that the individuals of *G. silaburensis* (16S: n = 10; CO1: n = 9) form one clade with 95% BS and 98% PP, the sister group of *G. bauensis*.

######### Discussion.

*Georissa silaburensis* was only found inside the cave entrance, with water flowing from the cave roof, and approximately less than 50% light penetration. In shell shape and reduced sculpture, it resembles another cave specialist, *G. filiasaulae*.

######## Georissa bauensis sp. n.

Taxon classificationAnimaliaORDOFAMILIA

http://zoobank.org/6E333906-75DD-4055-BD32-578E0D651E1F

######### Type locality.

Wind Cave Passage 3, Wind Cave Nature Reserve, Bau, Sarawak, Malaysia (01°24.81'N, 110°08.17'E).

######### Type material.

*Holotype*. Wind Cave Passage 3, Wind Cave Nature Reserve, Bau, Sarawak, Malaysia (01°24.81'N, 110°08.17'E): MZU/MOL 17.89 (leg. MZ Khalik). *Paratypes*. Wind Cave Passage 3, Wind Cave Nature Reserve, Bau, Sarawak, Malaysia (01°24.81'N, 110°08.17'E): MZU/MOL 16.01 (25), MZU/MOL 16.02 (>50). Gunung Podam, near Sungai Ayup, Kampung Bogag, Bau, Sarawak, Malaysia (01°21.15'N, 110°03.57'E): MZU/MOL 16.03 (5).

######### Etymology.

Named after the district of Bau, Sarawak, Malaysia, where the type locality Wind Cave Nature Reserve is located.

######### Description.

*Protoconch*. Color: red. Sculpture pattern: meshed – rounded or irregular mesh shape. Mesh width: 12–22 µm. *Teleoconch*. Color: orange to red. First whorl: shouldered, flat both above and below the shoulder. Subsequent whorls: convex shoulder and more rounded at the periphery. SH: 1.16–1.62 mm, SW: 1.06–1.30 mm, SI: 1.02–1.25. Total number of whorls: 2–2 ½. *Shell sculpture*. Radial sculpture: absent, only weak growth lines are visible. Spiral sculpture: present, weakly to strongly sculpted, continuous to discontinuous ribs, more prominent at the periphery. Scales: two to three major spiral series of low and small diagonal scales, regularly spaced, the upper series always stronger than the lower ones, scale series irregularly spaced. *Aperture*. Shape: rounded and tilted below. Basal side: rounded, angular at the columellar region. Parietal side: straight. AH: 0.57–0.78 mm, AW: 0.69–0.86 mm, AI: 0.74–0.96. *Holotype dimension*. SH: 1.16 mm, SW: 1.06 mm, AH: 0.58 mm, AW: 0.70 mm.

**Figure 14.**
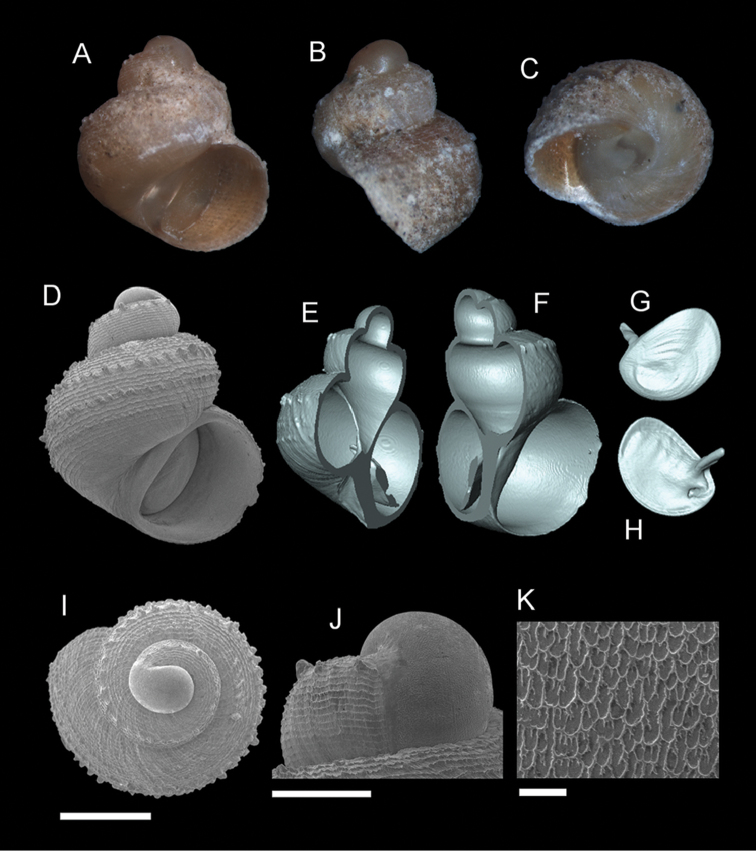
*Georissa bauensis* sp. n. **A–C** Holotype: MZU/MOL 17.89 **D–K** Paratypes: MZU/MOL 16.03. **A, D** Shell apertural view **B** Shell side view **C** Shell rear view **E–F** Shell cross-section from 3D model **G–H** Operculum frontal and ventral view **I** Shell top view **J** Protoconch side view **K** Close up of protoconch from top at 1000× magnification. Scale bars: 500 µm (**A–I**); 200 µm (**J**); 10 µm (**K**).

######### Cross diagnosis.

*Georissa bauensis* is very similar to *G. kobelti* (although not closely related phylogenetically), in terms of general shell shape and spiral scale characters. However, *G. bauensis* is sufficiently variable to include specimens that are more similar to *G. hosei* and *G. scalinella*. Furthermore, *G. bauensis* has more strongly sculpted scales than *G. hosei*, and a more rounded and convex shell than *G. scalinella*.

######### Distribution.

Known from Gunung Podam and Wind Cave Nature Reserve, Bau, Sarawak.

######### Molecular analysis.

ML and Bayesian analyses resolve all individuals of *G. bauensis* (16S: n = 13; CO1: n = 8) as a monophyletic group with 99% BS and 100% PP, the sister group of *G. silaburensis*.

######## Georissa pyrrhoderma

Taxon classificationAnimaliaORDOFAMILIA

Thompson & Dance, 1983

Georissa pyrrhoderma Thompson & Dance, 1983: 123, fig. 64.
Georissa pyrrhoderma van Benthem-Jutting, in Beron 2015: 181.

######### Type locality.

Borneo, Sarawak, First Division, western side of Gunong Selabor, Semabang entrance to Lobang Batu Cave (00°55'N, 110°25'E).

######### Type material.

*Holotype*. Borneo, Sarawak, First Division, western side of Gunong Selabor, Semabang entrance to Lobang Batu Cave: UF36183 (not seen). *Paratypes*. Borneo, Sarawak, First Division, western side of Gunong Selabor, Semabang entrance to Lobang Batu Cave: UF 36184, UF 36185 (not seen).

######### Other materials.

Gunong Silabur, Serian, Sarawak, Malaysia (00°57.45'N, 110°30.20'E): MZU/MOL 17.09, MZU/MOL 17.10, MZU/MOL 17.11, MZU/MOL 17.12, MZU/MOL 17.13, MZU/MOL 17.14, MZU/MOL 17.15, MZU/MOL 17.16, MZU/MOL 17.17, MZU/MOL 17.18, MZU/MOL 17.19, MZU/MOL 17.20, MZU/MOL 17.21, MZU/MOL 17.22, MZU/MOL 17.23, MZU/MOL 17.24.

######### Description.

*Protoconch*. Color: red to brown. Sculpture pattern: smooth to meshed, with ellipsoid mesh shape. Mesh width: 11–26 µm. *Teleoconch*. Color: brown to red. First whorl: shouldered, slightly curved above the shoulder, flat, cylindrical below the shoulder. Subsequent whorls: initially shouldered, but soon grading into uniformly rounded and quickly expanding whorls, with a deeply impressed suture; number of whorls: 2 ¼-2 ½. SH: 1.16–1.31 mm, SW: 1.12–1.20 mm, SI: 1.03–1.09. *Shell sculpture*. Radial sculpture: absent, only weak to strong growth lines are visible. Spiral sculpture: present, strong spiral sculpture. Scales: a single series of low, small and acute, unevenly spaced scales above the periphery, occasionally, in the vicinity of the aperture, subordinate series of minute scales accompany the major series. *Aperture*. Shape: rounded, tilted below the palatal side. Basal side: rounded, strongly angular at the columellar region. Parietal side: straight, palatal edge attached to the body whorl. AH: 0.58–0.63 mm, AW: 0.75–0.85 mm, AI: 0.73–0.81.

**Figure 15.**
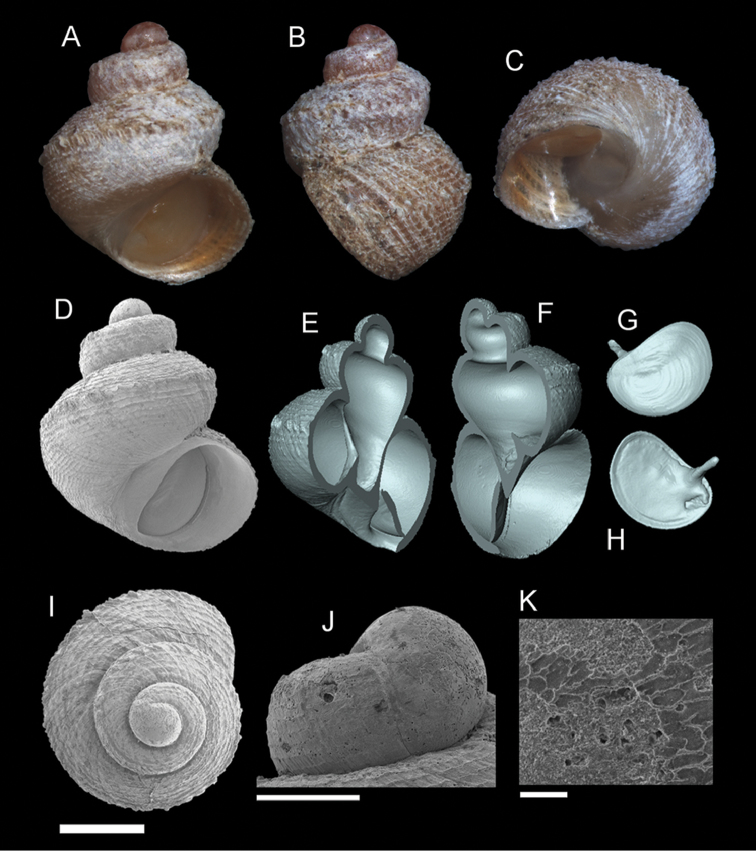
*Georissa pyrrhoderma* Thompson & Dance, 1983. **A–C** MZU/MOL 17.10 **D–K** MZU/MOL 17.09. **A, D** Shell apertural view **B** Shell side view **C** Shell rear view **E–F** Shell cross-section from 3D model **G–H** Operculum frontal and ventral view **I** Shell top view **J** Protoconch side view **K** Close up of protoconch from top at 1000× magnification. Scale bars: 500 µm (**A–I**); 200 µm (**J**); 10 µm (**K**).

######### Cross diagnosis.

*Georissa pyrrhoderma* has a shell habitus that is similar to *G. kobelti*, *G. hosei*, and *G. sepulutensis*. The latter two species are high variable and are morphologically, especially in sculpture, closely related to *G. pyrrhoderma*. Therefore, *G. pyrrhoderma* is nearly indistinguishable from certain forms of these other species.

######### Distribution.

Only known from the type locality, Gunung Silabur, Serian, Sarawak, Malaysia.

######### Molecular analysis.

In the ML and Bayesian analyses, all *G. pyrrhoderma* (16S: n = 28; CO1: n = 26) individuals group together in one clade with 99% BS and 100% PP. Its sister clade is *G. scalinella* + *G. kinabatanganensis*.

######### Discussion.

In the original description, Thompson and Dance (1983) did not compare *G. pyrrhoderma* with members of their *hosei*-group (which our molecular analyses show it belongs in). Instead, they considered it allied to *G. borneensis*. Possibly this misalignment was caused by the fact that the type specimens appear to lack the series of scales that is present on most of the specimens we collected. Nonetheless, given the restricted range of collection localities at Gunung Silabur and the degree of variability in our material, we consider our and Thompson and Dance’s material as conspecific. The paratypes specimen NHMUK 1984005 (Semabang entrance to Lobang Batu Cave, W. side of Gunong Selabor, 1^st^. Div., Sarawak, Malaysia: seen) is *G. silaburensis*.

######## Georissa kinabatanganensis sp. n.

Taxon classificationAnimaliaORDOFAMILIA

http://zoobank.org/A952A54F-D486-4C27-AFCA-8E7020E41ADA

######### Type locality.

Bukit Keruak, near Kinabatangan river, Sandakan, Sabah, Malaysia (05°31.385'N, 118°17.113'E).

######### Type material.

*Holotype*. Bukit Keruak, near Kinabatangan river, Sandakan, Sabah, Malaysia (05°31.38'N, 118°17.11'E): BOR/MOL 13921 (leg. M Schilthuizen). *Paratypes*. Bukit Keruak, near Kinabatangan river, Sandakan, Sabah (05°31.38'N, 118°17.11'E): MZU/MOL 17.26 (>50). BOR/MOL 1458, BOR/MOL 11656, BOR/MOL 11665, BOR/MOL 11711, BOR/MOL 13871. Batu Pangi, Sabah: BOR/MOL 1455. Batu Tomanggong, Sabah: BOR/MOL 1456, BOR/MOL 1457, BOR/MOL 10530.

######### Etymology.

Named after the district of Kinabatangan, Sabah, Malaysia, where the type locality Bukit Keruak is located.

######### Description.

*Protoconch*. Color: orange. Sculpture pattern: smooth to meshed – rounded to undefined mesh pattern. Mesh width: 14–21 µm. *Teleoconch*. Color: orange. First whorl: flat and angular at the shoulder. Subsequent whorls: angular, slightly rounded at the periphery, with number of whorls: 2–2 ¼. SH: 1.00–1.32 mm, SW: 1.13–1.37 mm, SI: 0.85–0.99. *Shell sculpture*. Radial sculpture: absent, only weak to strong growth lines are visible. Spiral sculpture: present, and strongly sculpted, with continuous to discontinuous ribbings. Scales: two series of diagonal vertical scales, widely spaced in between, both series are strongly sculpted, broad, and the scales are regularly placed. *Aperture*. Shape: oval. Basal side: rounded, angular at the columellar region. Parietal side: straight, palatal edge attached to the body whorl. AH: 0.54–0.66 mm, AW: 0.75–0.86 mm, AI: 0.65–0.80. *Holotype dimension*. SH: 1.00 mm, SW: 1.18 mm, AH: 0.54 mm, AW: 0.78 mm.

**Figure 16.**
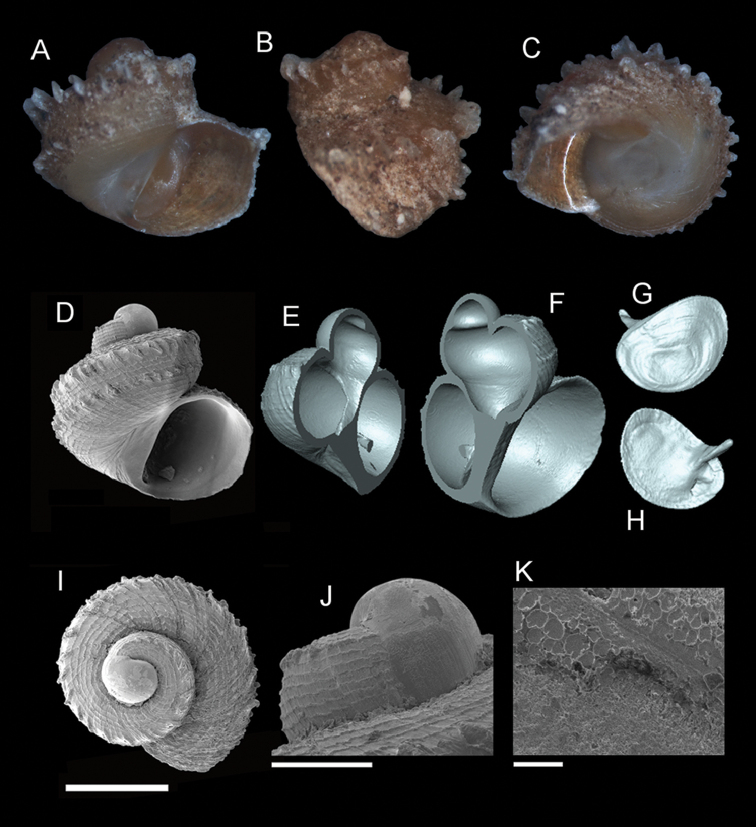
*Georissa kinabatanganensis* sp. n. **A–C** Holotype: BOR/MOL 13921 **D–K** Paratypes: MZU/MOL 17.26. **A, D** Shell apertural view **B** Shell side view **C** Shell rear view **E–F** Shell cross-section from 3D model **G–H** Operculum frontal and ventral view **I** Shell top view **J** Protoconch side view **K** Close up of protoconch from top at 1000× magnification. Scale bars: 500 µm (**A–I**); 200 µm (**J**); 10 µm (**K**).

######### Cross diagnosis.

*Georissa kinabatanganensis* has less variation in shell sculpture compared with *G. hosei* and *G. scalinella*. *G. kinabatanganensis* has two series of acutely projected scales on the whorls. In some cases, the second scale series is weaker than the first, and creates a series of nodular structures at the periphery. Often the shell is wider than high, which gives it a flattened appearance. In addition, *G. kinabatanganensis* has widely spaced between the scale series, similar to *G. muluensis*.

######### Distribution.

Known from Bukit Keruak, Batu Tomanggong, and Pangi, in the region of Kinabatangan, Sabah.

######### Molecular analysis.

RAxML and Bayesian analyses show *G. kinabatanganensis* (16S: n = 6; CO1: n = 6) forming a clade with 97% BS and 97% PP and as a sister clade to *G. sepulutensis*.

######### Discussion.

*Georissa kinabatanganensis* is the only species in “scaly group” *Georissa* to have a flat shell habitus, all examined individuals have a shell that is broader than high.

######## Georissa sepulutensis sp. n.

Taxon classificationAnimaliaORDOFAMILIA

http://zoobank.org/7D2EFD37-B493-4DE4-B219-4AA94BF2BD73

Georissa scalinella van Benthem-Jutting: Schilthuizen et al. 2005: 134-135 (**non**G. scalinella van Benthem-Jutting, 1966).

######### Type locality.

Sepulut valley, Gua Pungiton near Kg. Labang, Sabah, Malaysia (04°42.41'N, 116°36.04'E).

######### Type material.

*Holotype*. Sepulut valley, Gua Pungiton near Kg. Labang, Sabah, Malaysia (04°42.41'N, 116°36.04'E); BOR/MOL 13922 (leg. M Schilthuizen). *Paratypes*. Simbaluyon limestone hill, Sabah: RMNH/MOL 333905 (18), RMNH/MOL 333982 (23), RMNH/MOL 334006 (7). Tinahas, Sabah: RMNH/MOL 333984 (>50), RMNH/MOL 334013 (>50). Sepulut valley, Gua Sanaron, Sabah (04°42.05'N, 116°36.01'E): BOR/MOL 36, BOR/MOL 39, BOR/MOL 13870 (1). Sepulut Valley, Gua Pungiton, Sabah (04°42.41'N, 116°36.04'E): BOR/MOL 12278. Sepulut valley, Batu Punggul, Sabah: RMNH/MOL 187650, BOR/MOL.40. Baturong, Sabah: BOR/MOL 37.

######### Etymology.

Named after the town of Sepulut, Sabah, Malaysia, near which the type locality Gua Pungiton, as well as the other known localities, is located.

######### Description.

*Protoconch*. Color: red to brown. Sculpture: smooth to meshed – semi-oval mesh to undefined mesh pattern. Mesh width: 7–17 µm. *Teleoconch*. Color: red. First whorl: flat to rounded at the shoulder. Body whorl: rounded, with number of whorls: 2–2 ¾. SH: 1.11–1.52 mm, SW: 1.11–1.37 mm, SI: 0.94–1.07. *Shell sculpture*. Radial sculpture: absent, only weak growth lines are visible. Spiral sculpture: present, and strongly sculpted. Scales: a series of pointed vertical scales, acute and highly projected, and regularly spaced. *Aperture*. Shape: oval and tilted below. Basal side: rounded, angular at the columellar region. Parietal side: straight, palatal edge attached to the body whorl. AH: 0.62–0.81 mm, AW: 0.76–0.96 mm, AI: 0.72–0.87. *Holotype dimension*. SH: 1.34 mm, SW: 1.23 mm, AH: 0.65 mm, AW: 0.82 mm.

**Figure 17.**
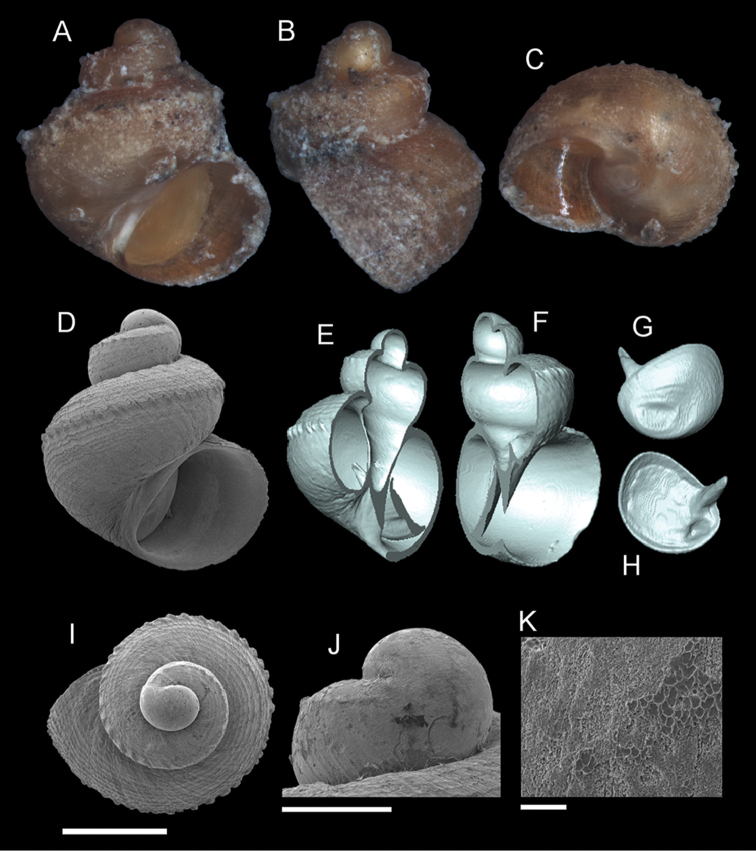
*Georissa sepulutensis* sp. n. **A–C** Holotype: BOR/MOL 13922 **D–K** Paratypes: BOR/MOL 12278. **A, D** Shell apertural view **B** Shell side view **C** Shell rear view **E–F** Shell cross-section from 3D model **G–H** Operculum frontal and ventral view **I** Shell top view **J** Protoconch side view **K** Close up of protoconch from top at 1000× magnification. Scale bars: 500 µm (**A–I**); 200 µm (**J**); 10 µm (**K**).

######### Cross diagnosis.

Unlike *Georissa kinabatanganensis*, *G. sepulutensis* has a series of scales only at the shoulder, which makes it resemble in habitus and scale characters *G. pyrrhoderma* from Gunung Silabur, Sarawak.

######### Distribution.

Distributed in the Sepulut Valley, Sabah; known from the following limestone localities: Simbaluyon, Sanaron, Tinahas, and Pungiton.

######### Molecular analysis.

ML and Bayesian analyses show *G. sepulutensis* (16S: n = 10; CO1: n = 2) as two clades with 93% BS and 97% PP, and as the sister species to *G. kinabatanganensis* sp. n.

######### Discussion.

*Georissa sepulutensis* and *G. kinabatanganensis* were previously included in *G. scalinella* (van Benthem-Jutting, 1966). Based on the genetic and morphological distinctness, we here consider them as separate species.

## Supplementary Material

XML Treatment for Georissa scalinella

XML Treatment for Georissa saulae

XML Treatment for Georissa hosei

XML Treatment for Georissa anyiensis

XML Treatment for Georissa muluensis

XML Treatment for Georissa hadra

XML Treatment for Georissa kobelti

XML Treatment for Georissa niahensis

XML Treatment for Georissa silaburensis

XML Treatment for Georissa bauensis

XML Treatment for Georissa pyrrhoderma

XML Treatment for Georissa kinabatanganensis

XML Treatment for Georissa sepulutensis

## Supplementary materials

Supplementary material 1 An overview of scanning parameters of each examined "scaly" *Georissa* Data type: Table in MS .docx file This is an open access article distributed under the terms of the Creative Commons Attribution License (CC BY 4.0), which permits unrestricted use, distribution, and reproduction in any medium, provided the original author and source are credited.Mohd Zacaery Khalik, Kasper Hendriks, Jaap J. Vermeulen, Menno Schilthuizen

Supplementary material 2 "Scaly" *Georissa* partitioning based on ABGD species delimitation Data type: Images embedded in MS .docx This is an open access article distributed under the terms of the Creative Commons Attribution License (CC BY 4.0), which permits unrestricted use, distribution, and reproduction in any medium, provided the original author and source are credited.Mohd Zacaery Khalik, Kasper Hendriks, Jaap J. Vermeulen, Menno Schilthuizen

Supplementary material 3
PTP species delimitation of "scaly" *Georissa* Data type: Image files .png and .svg, and .txt in .rar format This is an open access article distributed under the terms of the Creative Commons Attribution License (CC BY 4.0), which permits unrestricted use, distribution, and reproduction in any medium, provided the original author and source are credited.Mohd Zacaery Khalik, Kasper Hendriks, Jaap J. Vermeulen, Menno Schilthuizen

Supplementary material 4 Shell measurement of "scaly" *Georissa* Data type: Shell measurement in MS .xlsx format This is an open access article distributed under the terms of the Creative Commons Attribution License (CC BY 4.0), which permits unrestricted use, distribution, and reproduction in any medium, provided the original author and source are credited.Mohd Zacaery Khalik, Kasper Hendriks, Jaap J. Vermeulen, Menno Schilthuizen

Supplementary material 5 Synoptic view of 13 species of "scaly" *Georissa*, and their 3D models Data type: Video .mp4 file This is an open access article distributed under the terms of the Creative Commons Attribution License (CC BY 4.0), which permits unrestricted use, distribution, and reproduction in any medium, provided the original author and source are credited.Mohd Zacaery Khalik, Kasper Hendriks, Jaap J. Vermeulen, Menno Schilthuizen

## References

[B1] Bandel K (2008) Operculum shape and construction of some fossil Neritimorpha (Gastropoda) compared to those of modern species of the subclass. Vita Malacologica 7: 19–36. http://www.paleoliste.de/bandel/bandel_2008.pdf

[B2] Beron P (2015) Comparative study of the invertebrate cave faunas of Southeast Asia and New Guinea. Historia Naturalis Bulgarica 21: 169–210. http://www.nmnhs.com/historia-naturalis-bulgarica/pdfs/000365000212015.pdf

[B3] Berry A (1966) Population structure and fluctuations in the snail fauna of a Malayan limestone hill. Proceedings of the Zoological Society of London 150(1): 11–27. 10.1111/j.1469-7998.1966.tb02996.x

[B4] Blanford WT (1864) XLII.-On the classification of the Cyclostomacea of Eastern Asia. Journal of Natural History 13(78): 441–465. 10.1080/00222936408681635

[B5] Clements R Ng PK Lu XX Ambu S Schilthuizen M Bradshaw CJ (2008) Using biogeographical patterns of endemic land snails to improve conservation planning for limestone karsts. Biological Conservation 141(11): 2751–2764. 10.1016/j.biocon.2008.08.011

[B6] Clements R Sodhi NS Schilthuizen M Ng PK (2006) Limestone karsts of Southeast Asia: imperiled arks of biodiversity. BioScience 56(9): 733–742. 10.1641/0006-3568(2006)56[733:LKOSAI]2.0.CO;2

[B7] Edgar RC (2004) MUSCLE: multiple sequence alignment with high accuracy and high throughput. Nucleic Acids Research 32(5): 1792–1797. 10.1093/nar/gkh340 15034147 PMC390337

[B8] Gredler PV (1902) Zur Conchylien-Fauna von Borneo and Celebes. Nachrichtsblatt der Deutschen Malakozoologischen Gesellschaft: 53–64. https://www.biodiversitylibrary.org/page/15598792#page/459/mode/1up

[B9] Godwin-Austen HH (1889) On a collection of land-shells made in Borneo by Mr. A. Everett with supposed new species. Part 1. Proceedings of the Zoological Society of London: 332–355. https://www.biodiversitylibrary.org/page/30866786#page/434/mode/1up

[B10] Haase M Schilthuizen M (2007) A new *Georissa* (Gastropoda: Neritopsina: Hydrocenidae) from a limestone cave in Malaysian Borneo. Journal of Molluscan Studies 73(3): 215–221. 10.1093/mollus/eym020

[B11] Hoang DT Chernomor O von Haeseler A Minh BQ Vinh LS (2017) UFBoot2: Improving the Ultrafast Bootstrap Approximation. Molecular Biology and Evolution 35(2): 518–522. 10.1093/molbev/msx281 PMC5850222 29077904

[B12] Huelsenbeck JP Ronquist F (2001) MRBAYES: Bayesian inference of phylogenetic trees. Bioinformatics 17(8): 754–755. 10.1093/bioinformatics/17.8.754 11524383

[B13] Kalyaanamoorthy S Minh BQ Wong TKF von Haeseler A Jermiin LS (2017) ModelFinder: fast model selection for accurate phylogenetic estimates. Nature Methods 14(6): 587–589. 10.1038/nmeth.4285 28481363 PMC5453245

[B14] Kumar S Stecher G Tamura K (2016) MEGA7: Molecular Evolutionary Genetics Analysis version 7.0 for bigger datasets. Molecular Biology and Evolution 33(7): 1870–1874. 10.1093/molbev/msw054 27004904 PMC8210823

[B15] Liew TS Vermeulen JJ Marzuki ME Schilthuizen M (2014) A cybertaxonomic revision of the micro-landsnail genus *Plectostoma* Adam (Mollusca, Caenogastropoda, Diplommatinidae), from Peninsular Malaysia, Sumatra and Indochina. ZooKeys 393: 1–107. 10.3897/zookeys.393.6717 PMC3974427 24715783

[B16] Liew TS Schilthuizen M (2016) A method for quantifying, visualising, and analysing gastropod shell form. PloS One 11(6): e0157069. 10.1371/journal.pone.0157069 PMC4900530 27280463

[B17] Mayr E (1942) Systematics and the origin of species, from the viewpoint of a zoologist. Harvard University Press. http://krishikosh.egranth.ac.in/bitstream/1/2050669/1/IARInew-00187.pdf

[B18] Nguyen LT Schmidt HA von Haeseler A Minh BQ (2014) IQ-TREE: a fast and effective stochastic algorithm for estimating maximum-likelihood phylogenies. Molecular Biology and Evolution 32(1): 268–274. 10.1093/molbev/msu300 25371430 PMC4271533

[B19] Osikowski A Hofman S Georgiev D Rysiewska A Falniowski A (2017) Unique, ancient stygobiont clade of Hydrobiidae (Truncatelloidea) in Bulgaria: the origin of cave fauna. Folia Biologica (Kraków) 65(2): 79–93. 10.3409/fb65_2.79

[B20] Phung CC Yu FTY Liew TS (2017) A checklist of land snails from the west coast islands of Sabah, Borneo (Mollusca, Gastropoda). ZooKeys 673: 49–104. 10.3897/zookeys.673.12422 PMC5523196 28769672

[B21] Puillandre N Lambert A Brouillet S Achaz G (2012a) ABGD, Automatic Barcode Gap Discovery for primary species delimitation. Molecular Ecology 21(8): 1864–1877. 10.1111/j.1365-294X.2011.05239.x 21883587

[B22] Puillandre N Modica MV Zhang Y Sirovich L Boisselier MC Cruaud C Holford M Samadi S (2012b) Large‐scale species delimitation method for hyperdiverse groups. Molecular Ecology 21(11): 2671–2691. 10.1111/j.1365-294X.2012.05559.x 22494453

[B23] Rundell RJ (2008) Cryptic diversity, molecular phylogeny and biogeography of the rock-and leaf litter-dwelling land snails of Belau (Republic of Palau, Oceania). Philosophical Transactions of the Royal Society B: Biological Sciences 363(1508): 3401–3412. 10.1098/rstb.2008.0110 PMC2607371 18765361

[B24] Saul M (1967) Shell collecting in the limestone caves of Borneo. Sabah Society Journal 3: 105–110.

[B25] Schilthuizen M (2011) Community ecology of tropical forest snails 30 years after Solem. Contributions to Zoology 80(1): 1–15. http://www.ctoz.nl/vol80/nr01/a01

[B26] Schilthuizen M Cabanban AS Haase M (2005) Possible speciation with gene flow in tropical cave snails. Journal of Zoological Systematics and Evolutionary Research 43(2): 133–138. 10.1111/j.1439-0469.2004.00289.x

[B27] Schilthuizen M Gittenberger E (1996) Allozyme variation in some Cretan Albinaria (Gastropoda): paraphyletic species as natural phenomena. In: Taylor JD (Ed.) Origin and Evolutionary Radiation of the Mollusca. Oxford University Press Inc., New York, 301–311.

[B28] Schilthuizen M Liew TS Elahan BB Lackman-Ancrenaz I (2005) Effects of karst forest degradation on pulmonate and prosobranch land snail communities in Sabah, Malaysian Borneo. Conservation Biology 19(3): 949–954. 10.1111/j.1523-1739.2005.00209.x

[B29] Schilthuizen M Rutten EJM Haase M (2012) Small-scale genetic structuring in a tropical cave snail and admixture with its above-ground sister species. Biological Journal of the Linnean Society 105(4): 727–740. 10.1111/j.1095-8312.2011.01835.x

[B30] Schilthuizen M Van Til A Salverda M Liew TS James SS Elahan BB Vermeulen JJ (2006) Microgeographic evolution of snail shell shape and predator behavior. Evolution 60(9): 1851–1858. 10.1554/06-114.1 17089969

[B31] Smith EA (1893) Descriptions of new species of land-shells from Borneo. Zoological Journal of the Linnean Society 24(154): 341–352. 10.1111/j.1096-3642.1893.tb02488.x

[B32] Smith EA (1895) On a collection of land-shells from Sarawak, British North Borneo, Palawan, and other neighboring islands. In Proceedings of the Zoological Society of London 63: 97–127. https://www.biodiversitylibrary.org/item/97158#page/135/mode/1up

[B33] Stamatakis A (2014) RAxML version 8: a tool for phylogenetic analysis and post-analysis of large phylogenies. Bioinformatics 30(9): 1312–1313. 10.1093/bioinformatics/btu033 24451623 PMC3998144

[B34] Thompson FG Dance SP (1983) Non-marine mollusks of Borneo. II Pulmonata: Pupillidae, Clausiliidae. III Prosobranchia: Hydrocenidae, Helicinidae. Bulletin of the Florida State Museum Biological Sciences 29(3): 101–152. https://www.floridamuseum.ufl.edu/files/2114/7180/1931/Vol-29-No-3.PDF

[B35] Tongkerd P Lee T Panha S Burch JB O’Foighil D (2004) Molecular phylogeny of certain Thai gastrocoptine micro land snails (Stylommatophora: Pupillidae) inferred from mitochondrial and nuclear ribosomal DNA sequences. Journal of Molluscan Studies 70(2): 139–147. 10.1093/mollus/70.2.139

[B36] van Benthem-Jutting WSS (1966) Two new species of Hydrocena (Neritacea) from Sabah, Borneo. Journal of Conchology 26: 39–41.

[B37] Vermeulen JJ Junau D (2007) Bukit Sarang (Sarawak, Malaysia), an isolated limestone hill with an extraordinary snail fauna. Basteria 71(4/6): 209–220. http://natuurtijdschriften.nl/record/597351

[B38] Vermeulen JJ Liew TS Schilthuizen M (2015) Additions to the knowledge of the land snails of Sabah (Malaysia, Borneo), including 48 new species. ZooKeys 531: 1–139. 10.3897/zookeys.531.6097 PMC4668911 26692803

[B39] Vermeulen JJ Whitten T (1998) Fauna Malesiana guide to the land snails of Bali. Backhuys Publishers, Leiden, The Netherlands.

[B40] Zhang J Kapli P Pavlidis P Stamatakis A (2013) A general species delimitation method with applications to phylogenetic placements. Bioinformatics 29(22): 2869–2876. 10.1093/bioinformatics/btt499 23990417 PMC3810850

[B41] Zilch A (1973) Die typen und typoide des Natur-Museums Senckenberg. Mollusca: Hydrocenidae. Archiv für Molluskenkunde 103(4/6): 263–272.

