# Supplementary material for: A molecular and conchological dissection of the “scaly” Georissa of Malaysian Borneo (Gastropoda, Neritimorpha, Hydrocenidae)
Source: Zookeys. 2018 Jul 9;(773):1–55. doi: 10.3897/zookeys.773.24878 (PMC6048177; doi:10.3897/zookeys.773.24878)
Supplement: Supplementary material 1 — An overview of scanning parameters of each examined "scaly" Georissa [file zookeys-773-001-s001.docx]

**Supplementary material 1**

Table S1.1 An overview of scanning parameters of each examined “scaly” *Georissa*.

| No. | Species | Filter type | Detector distance (mm) | Pixel size (µm) | Transmission  (%) | Exposure time (sec.) | Intensity |
| --- | --- | --- | --- | --- | --- | --- | --- |
| 1 | *G. anyiensis* n. sp. | LE2 | 30 | 3.0666 | 46-76 | 1.0 | 6000-9000 |
| 2 | *G. bauensis* n. sp. | LE2 | 30 | 2.2499 | 46-76 | 1.0 | 5500-7600 |
| 3 | *G. hadra* | LE1 | 25 | 4.1517 | 58-74 | 1.5 | 6400-9400 |
| 4 | *G. hosei* | LE2 | 30 | 2.6995 | 46-76 | 1.0 | 5700-8000 |
| 5 | *G. kobelti* | LE1 | 40 | 2.8914 | 58-74 | 1.5 | 5500-7500 |
| 6 | *G. muluensis* n. sp. | LE1 | 40 | 2.8914 | 58-74 | 1.5 | 6500-8600 |
| 7 | *G. niahensis* | LE1 | 30 | 3.3733 | 58-74 | 1.5 | 5900-10000 |
| 8 | *G. kinabatanganensis* n. sp. | LE2 | 40 | 2.2494 | 46-76 | 1.0 | 5800-8400 |
| 9 | *G. pyrrhoderma* | LE1 | 50 | 1.9962 | 58-78 | 1.0 | 5900-8300 |
| 10 | *G. sepulutensis* n. sp. | LE2 | 35 | 2.4534 | 46-76 | 1.0 | 6000-9000 |
| 11 | *G. saulae* | LE1 | 60 | 1.5574 | 63-90 | 2.0 | 7000-9500 |
| 12 | *G. scalinella* | LE2 | 30 | 2.6986 | 61-72 | 2.0 | 5300-8100 |
| 13 | *G. silaburensis* n. sp. | LE1 | 50 | 2.3240 | 58-74 | 1.0 | 5500-7300 |
