# Supplementary material for: A molecular and conchological dissection of the “scaly” Georissa of Malaysian Borneo (Gastropoda, Neritimorpha, Hydrocenidae)
Source: Zookeys. 2018 Jul 9;(773):1–55. doi: 10.3897/zookeys.773.24878 (PMC6048177; doi:10.3897/zookeys.773.24878)
Supplement: Supplementary material 2 — "Scaly" Georissa partitioning based on ABGD species delimitation [file zookeys-773-001-s002.docx]

**Supplementary material 2**


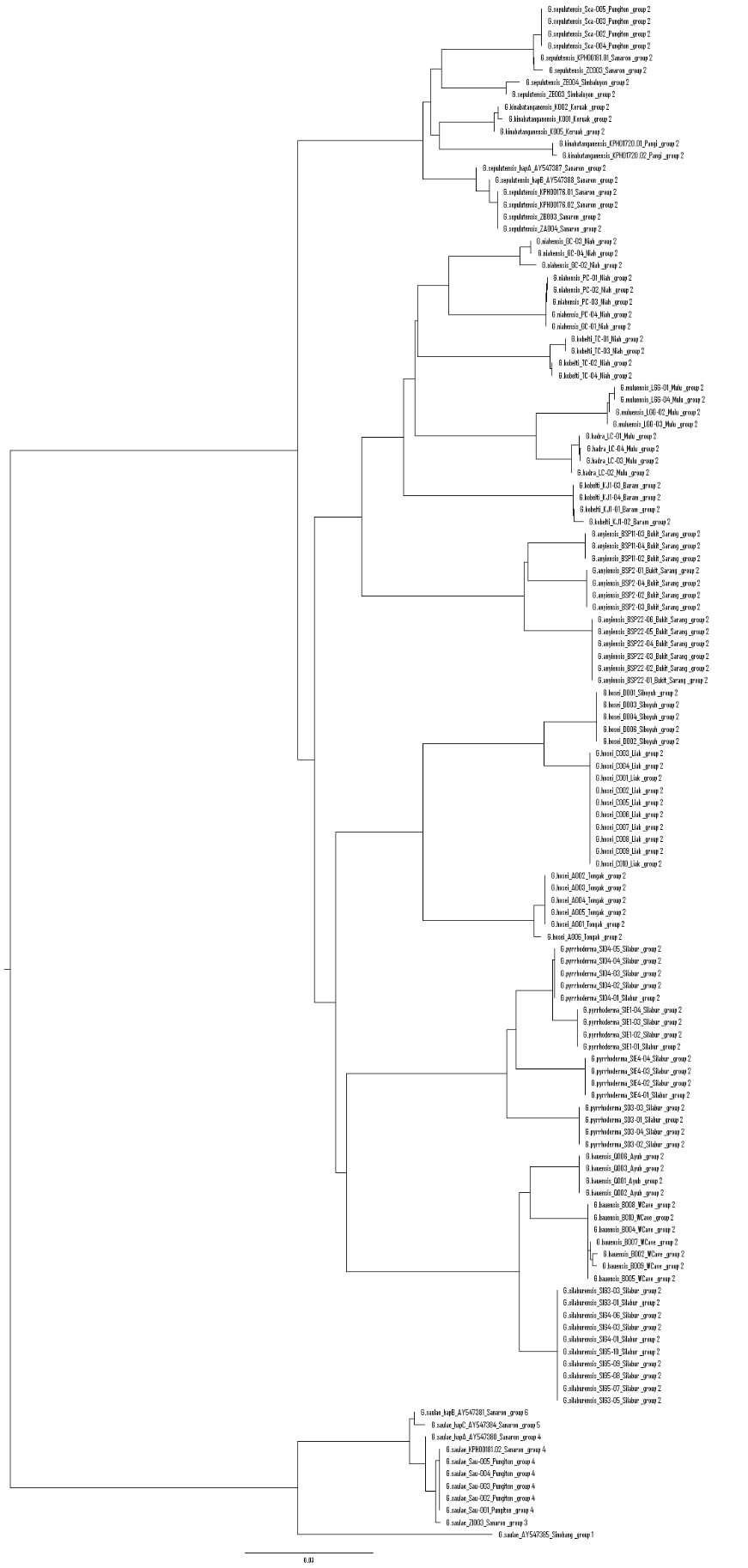


**Figure S2.1** “Scaly” *Georissa* partitioning based on ABGD analysis at the lowest intraspecific divergence by using 16S mtDNA sequence alignment.


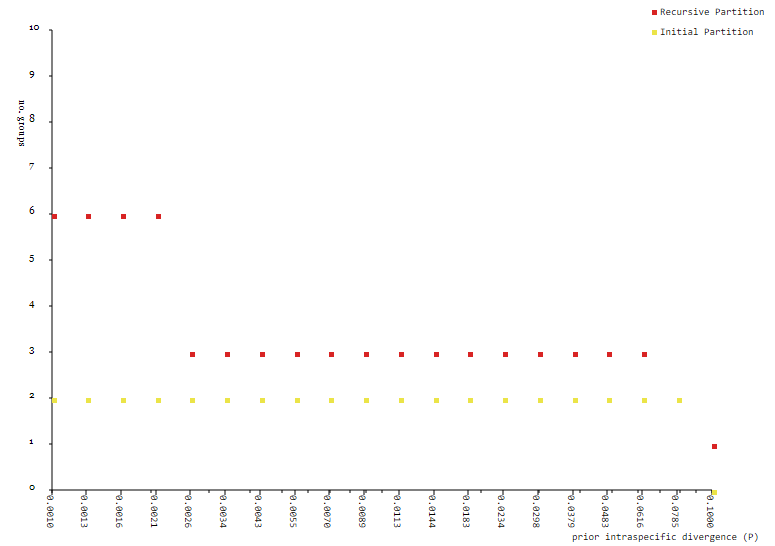


**Figure S2.2** Number of groups from both initial and recursive partitioning from ABGD analysis at different level of intraspecific divergence.
